# Interactions between genetic predisposition to obesity, insulin resistance and type 2 diabetes risk, and food or beverage intake for incident type 2 diabetes: European Prospective Investigation into Cancer and Nutrition (EPIC) InterAct case–cohort study

**DOI:** 10.1016/j.ajcnut.2026.101198

**Published:** 2026-01-16

**Authors:** Sherly X Li, Fumiaki Imamura, Stephen J Sharp, Matthias B Schulze, Ju-Sheng Zheng, Pilar Amiano, Eva Ardanaz, Manuela M Bergmann, Maria-Dolores Chirlaque, Guy Fagherazzi, Paul W Franks, Sara Grioni, Daniel B Ibsen, Paula Jakszyn, Ingegerd Johansson, Verena A Katzke, Nasser Laouali, Francesca R Mancini, Kim Overvad, Domenico Palli, Salvatore Panico, Daniel Redondo-Sánchez, Fulvio Ricceri, Olov Rolandsson, Bernard Srour, Anne Tjønneland, Tammy YN Tong, Yvonne T van der Schouw, Elio Riboli, Claudia Langenberg, Nita G Forouhi, Nick J Wareham

**Affiliations:** 1 MRC Epidemiology Unit, University of Cambridge School of Clinical Medicine, Institute of Metabolic Science, Cambridge Biomedical Campus Cambridge, Cambridge, United Kingdom; 2 Murdoch Children’s Research Institute, The Royal Children’s Hospital, Melbourne, VIC, Australia; 3 Centre for Epidemiology and Biostatistics, University of Melbourne, Melbourne, VIC, Australia; 4 Department of Molecular Epidemiology, German Institute of Human Nutrition Potsdam-Rehbruecke, Nuthetal, Germany; 5 German Center for Diabetes Research (DZD), Neuherberg, Germany; 6 Institute of Nutritional Science, University of Potsdam, Potsdam, Germany; 7 School of Life Sciences, Westlake University, Hangzhou, Zhejiang, China; 8 Ministry of Health of the Basque Government, Sub Directorate for Public Health and Addictions of Gipuzkoa, San Sebastián, Spain; 9 Epidemiology of Chronic and Communicable Diseases GroupBiodonostia Health Research Institute, San Sebastián, Spain; 10 Consortium for Biomedical Research in Epidemiology and Public Health (CIBER Epidemiología y Salud Pública), Madrid, Spain; 11 Public Health Institute of Navarra, IdiSNA, Pamplona, Spain; 12 IdiSNA, Navarra Institute for Health Research, Calle de Irunlarrea, Pamplona, Navarra, Spain; 13 Human Study Center, German Institute of Human Nutrition Potsdam-Rehbruecke, Nuthetal, Germany; 14 Department of Epidemiology, Murcia Regional Health Council, IMIB-Arrixaca, Murcia, Spain; 15 Deep Digital Phenotyping Research Unit, Department of Precision Health, Luxembourg Institute of Health, Strassen, Luxembourg; 16 Center of Epidemiology and Population Health UMR 1018, Inserm, Paris South – Paris Saclay University, Gustave Roussy Institute, Villejuif, France; 17 Department of Clinical Sciences, Clinical Research Center, Skåne University Hospital, Lund University, Malmö, Sweden; 18 Department of Public Health and Clinical Medicine, Umeå University, Umeå, Sweden; 19 Epidemiology and Prevention Unit, Fondazione IRCCS Istituto Nazionale dei Tumori Milan, Milan, Italy; 20 Department of Public Health, Aarhus University, Aarhus C, Denmark; 21 Unit of Cardiovascular and Nutritional Epidemiology, Institute of Environmental Medicine, Karolinska Institute, Solna, Sweden; 22 Unit of Nutrition and Cancer, Cancer Epidemiology Research Programme, Catalan Institute of Oncology (ICO-IDIBELL), Barcelona, Spain; 23 Facultat Ciències Salut Blanquerna, Universitat Ramon Llull, Barcelona, Spain; 24 Department of Odontology, School of Dentistry, Cariology, Umeå University, Umeå, Sweden; 25 Division of Cancer Epidemiology, German Cancer Research Center (DKFZ), Heidelberg, Germany; 26 CESP, Faculty of Medicine – University Paris-South, Faculty of Medicine INSERM U1018, University Paris-Saclay, Villejuif, France; 27 Department of Cardiology, Aalborg University Hospital, Aalborg, Denmark; 28 Cancer Risk Factors and Life-Style Epidemiology Unit, Institute for Cancer Research, Prevention and Clinical Network – ISPRO, Villa delle Rose, Florence, Italy; 29 Dipartimento di Medicina Clinica e Chirurgia, Federico II University, Naples, Italy; 30 Escuela Andaluza de Salud Pública (EASP), Granada, Spain; 31 Instituto de Investigación Biosanitaria ibs.GRANADA, Granada, Spain; 32 Centre for Biostatistics, Epidemiology, and Public Health (C-BEPH), Department of Clinical and Biological Sciences, University of Turin, Orbassano (TO), Italy; 33 Department of Public Health and Clinical Medicine, Family Medicine, Umeå University, Umeå, Sweden; 34 Centre for Research in Epidemiology and Statistics, Université Sorbonne Paris Nord and Université Paris Cité, Bobigny, France; 35 Danish Cancer Society Research Center, Copenhagen, Denmark; 36 Cancer Epidemiology Unit, Nuffield Department of Population Health, University of Oxford, Oxford, United Kingdom; 37 Julius Center for Health Sciences and Primary Care, University Medical Center Utrecht, Utrecht University, Utrecht, The Netherlands; 38 School of Public Health, Imperial College London, Norfolk Place, London, United Kingdom; 39 Precision Healthcare University Research Institute, Queen Mary University of London, London, United Kingdom; 40 Computational Medicine, Berlin Institute of Health at Charité – Universitätsmedizin Berlin, Berlin, Germany

**Keywords:** diet, polygenic risk score, obesity, insulin resistance, diabetes, gene–diet interaction, effect modification, epidemiology, precision nutrition, nutritional epidemiology

## Abstract

**Background:**

Limited evidence exists for effect modification of genetic characteristics on the associations of food consumption and incident type 2 diabetes (T2D).

**Objectives:**

We aimed to investigate whether the food-T2D association would vary by genetic susceptibility to metabolic traits.

**Methods:**

We analyzed data from 9542 incident T2D cases and a subcohort of 12,477 participants nested within the 340,234-participant cohort recruited in 1991–1998 and followed up for 10.9 y on average in 8 European countries. Polygenic risk scores (PRSs) for higher body mass index, insulin resistance, and T2D were constructed. Fifteen dietary variables potentially associated with T2D, obtained with cohort-specific self-reported dietary assessment, were examined: fruits, green leafy vegetables, root vegetables, wholegrains, rice, legumes, nuts and seeds, fermented dairy, red meat, processed meat, fish, eggs and egg products, sugar-sweetened beverages, coffee, and tea. A cross-product term between each PRS and each food/beverage was evaluated by genotyping chip and country with Prentice-weighted Cox regression for incident T2D, and stratum-specific estimates were meta analyzed, followed by Benjamini–Yekutieli multiple-testing correction.

**Results:**

Accounting for multiple tests of 3 PRSs × 15 dietary items, no evidence of statistical interaction was evident on either a multiplicative or additive scale, with exp(β for a multiplicative interaction) (95% confidence interval) ranging from 0.84 (0.64, 1.10) (root vegetables and PRS for T2D) to 1.45 (0.78–2.76) (fish and PRS for T2D).

**Conclusions:**

Genetic susceptibility to high-risk metabolic traits did not modify the diet-T2D associations in European populations. Acknowledging the limitations of current PRS-based methods to detect gene–diet interactions, research should continue into the potential for precision nutrition and tailored food-based dietary guidance for T2D prevention.

## Introduction

There is an escalating global healthcare and economic burden because of type 2 diabetes (T2D) [1] and health behaviors including diet constitute a key component of T2D prevention strategies [2,3]. Current nutritional guidelines emphasize consumption of, for example, wholegrains, fruit and vegetables, and a reduction in consumption of red and processed meats and sugar-sweetened beverages (SSBs) for the prevention of T2D [2,4,5]. A criticism of such guidelines is the assumption of homogenous dietary effects, for example, not accounting for potential individual variation by genetic background or other personal characteristics. To circumvent the criticism, “precision nutrition” has received attention, in keeping with the Precision Medicine Initiative on personalized or stratified approaches to disease prevention or management [6,7]. Precision nutrition aims to tailor dietary recommendations and nutritional interventions for the prevention and management of chronic disease based on individual characteristics including genetics.

For foods, some studies have reported, for example, that consuming wholegrains and coffee was associated with T2D risk differently depending on *TCF7L2* variants [[8], [9], 10]]. Polygenic risk scores (PRSs), composed of multiple single-nucleotide variants, are also of interest because they may better reflect the polygenic nature of T2D than single-nucleotide variants [11]. We previously reported that PRSs for susceptibility to T2D, insulin resistance (IR), and higher BMI (in kg/m^2^) did not modify the association between macronutrient intake and incidence of T2D [12]. A 15-cohort meta-analysis showed no evidence for the interaction between fatty acid intakes and PRS for T2D risk for T2D incidence [13]. Interactions between SSB intake and BMI PRS have been replicated for adiposity traits in studies from the United States, Denmark, and Sweden [[14], [15], [16]]. A single cross-sectional study in China indicated that the effect of PRS for T2D on T2D risk would be lower when fruit consumption was high [17], whereas no food-PRS interaction for T2D incidence was observed when analyzing food or beverage consumption in Sweden and the United Kingdom [18,19]. To date, there has been still limited evidence for interaction between individual food groups and genetic susceptibility evaluated with PRSs for BMI, IR, and T2D [10,[17], [18], [19], [20], [21], [22]]. Thus, with updated PRSs for those phenotypes [[23], [24], [25]] and multinational dietary data standardized across European countries, we aimed to test the hypotheses of multiplicative and additive interactions between major food groups and PRSs for BMI, IR, and T2D on incident T2D.

## Methods

Described in detail previously [26], EPIC-InterAct is a case–cohort study across 8 European countries (France, Italy, Spain, the United Kingdom, the Netherlands, Germany, Denmark, and Sweden), nested within the European Prospective Investigation into Cancer and Nutrition (EPIC). From among 340,234 eligible participants with 3.99 million person-years of follow-up who had available stored blood, incident T2D cases were ascertained and verified from 2 or more independent sources including self-report, linkage to primary care registers, secondary-care registers, medication use, hospital admission, or mortality data [26] (Supplemental Figure 1). A random subcohort of 16,835 participants was selected, for which we estimated statistical power of >90% under various scenarios to detect a gene-environment interaction after excluding those who met exclusion criteria [26]. We excluded individuals who met one of the exclusion criteria; prevalent T2D at baseline (1991–1998), T2D cases observed after the censoring date (31 December 2007) or unknown T2D status (*n* = 6206); without genetic, dietary, and covariate data (*n* = 5287, 592, and 463, respectively). This exclusion left 21,437 participants for the current analysis: 9542 T2D cases (582 T2D cases in the subcohort); and 11,895 subcohort noncases (*n* in the total subcohort = 12,477). As per a case–cohort design, those in the subcohort also contributed to a small number of cases, accounted for in the statistical analysis [26]. All participants gave written informed consent, and ethical approval was obtained at each participating research center.

### Genotyping and genetic risk scores

Biological samples were stored in the International Agency for Research on Cancer, Lyon, France, or local biobanks (Supplemental Text) [26]. A DNA sample from buffy coat from each eligible participant was extracted and genotyped on the Illumina 660W-Quad BeadChip (*n* = 8821) or Illumina HumanCore Exome chip arrays (*n* = 12,616) and imputed to the Haplotype Reference Consortium using IMPUTE v2.3.2. We generated 3 separate weighted PRSs for BMI, IR, and T2D as the primary genetic variables. For each of the traits, we calculated a product of number or imputed dose levels of each single-nucleotide polymorphism (SNP) (0–2) and its effect size (β coefficient) obtained from published meta-analyses of genome-wide association study (GWAS) results [11] and summed up the products. SNPs from loci reaching genome-wide significance for the respective traits that have been confirmed in published meta-analyses of studies in European populations were chosen (751 SNPs for BMI [23]; 53 for IR [24]; and 403 for T2D [25]). Unweighted PRSs for those traits were also calculated as the secondary genetic variables (weight = 1 for all SNPs).

As post hoc analyses, 247 SNPs were evaluated additionally for an interaction with 1 of the selected food variables, contributing to 1 or more of the 3 PRSs identified in the earlier consortia effort [24,27,28]. Those from earlier GWAS results were selected to reduce multiple test burdens, avoid variants with lower allele frequencies, and weaker average effects identified in later GWAS results (i.e., lower statistical power), and anticipate available genetic and functional information to be annotated to inform future candidate-gene work [29].

### Self-reported dietary intake

We assessed the habitual consumption of 15 food groups (foods and beverages; Supplemental Table 1) that were previously investigated for their associations with T2D in published systematic reviews and meta-analyses [3,30,31]. These included fruits, green leafy vegetables, root vegetables (not including potatoes), wholegrain cereal products and nonwhite breads, rice, legumes, nuts and seeds, fermented dairy, red meat, processed meat, fish, egg and egg products, SSBs, coffee, and tea. Validity of the instruments to capture habitual diet was assessed in each participating cohort [32,33]. Data on all 15 food groups were available in EPIC-InterAct, except for legume intake not assessed in Denmark where its levels would be generally low [34]. Dietary data on habitual consumption over the previous year was derived in g/d from country-specific food frequency questionnaires or dietary histories taken at baseline. Each food or beverage was scaled to an average portion size assumed to reflect a standard amount of consumption, according to the UK Food Standards Agency [35].

### Assessment of covariates

A baseline questionnaire was completed and provided information including age, sex, alcohol consumption, physical activity, highest education level, smoking status, medical history, and family history of diabetes. Anthropometric measures (including body weight and height) were obtained by trained researchers using standardized methods or self-reported in some centers (Oxford in the United Kingdom, centers in France). Details are described in the Supplemental Text.

### Statistical analyses

All analyses were conducted with Stata version 17 (StataCorp LP) and complete-case. Dietary exposures were analyzed as continuous variables (per portion/d) and winsorized at the 99th percentile. Each PRS was standardized with statistics from the subcohort. Each of 15 foods and beverages and 3 PRSs was evaluated for the association with incident T2D, using multivariable-adjusted Prentice-weighted Cox regression models [26] (Supplemental Text). The interactions between each dietary factor and each PRS were investigated in a gene-chip- and country-specific Cox regression model, including 15 dietary factors, 1 of the PRSs, and a cross-product term of the PRS and a single dietary factor. The model included age as the underlying timescale, sex, centers, total energy intake, physical activity (inactive, moderately inactive, moderately active, active), education (none, primary school, technical/professional, secondary school, longer education, including university), smoking (never, former, current smoker), alcohol consumption, and 15 foods and beverages; legume variable in Denmark was not included in the model. We further adjusted for BMI to minimize its confounding role, except when analyzing the BMI PRS. Gene-chip- and country-specific estimates of hazard ratios (HRs) per portion/d of each dietary exposure were pooled using random-effects meta-analysis.

Using the pooled parameter estimates, both multiplicative and additive interactions were tested [36,37]. Multiplicative interactions refer to deviations from the expected effect assuming the individual exposures multiply on a relative scale (HR), whereas additive interactions refer to deviations on an absolute scale (incidence difference). Additive interactions are particularly relevant for public health because they indicate the absolute excess risk attributable to the joint exposure, which can guide prioritization of interventions [36]. To account for multiple testing with 15 dietary exposures and 3 PRSs (2-sided α = 0.05), a significant threshold was adjusted with a Benjamini–Yekutieli approach that would be robust against multiple tests involving correlated dietary factors [38]. As additional aids, we projected quartile-quartile (QQ) plots of −log_10_(*P* values). We also stratified by a median of each of the 3 PRSs to enable visual comparisons in HRs for a diet-T2D association.

As further exploratory analyses, multiplicative interactions were examined for 247 individual SNPs as a potential effect-modifier for a diet–T2D association. The aforementioned Cox regression analysis was repeated by replacing a PRS with 1 of the SNPs [3705 times (=15 foods/beverages × 247 SNPs)], projecting QQ plots and applying a Benjamini–Yekutieli false discovery rate correction. These procedures were repeated twice with and without adjustment for BMI. For transparency for future biologically focused investigations, SNPs’ estimates with *P* values <0.05 were systematically matched with existing biological and epidemiological information available in the Ensembl database, the GWAS catalog, and the Genetic Association Database, queried in SNPnexus [39].

## Results

Participants were 52.4 y old (SD = 9.2 y) (Table 1) and followed up for 10.9 y on average. In adjusted analyses, higher PRS for each trait was associated with higher T2D incidence (Table 2). There were positive associations of habitual consumption of red and processed meat, SSBs, and green leafy vegetables with incident T2D and negative associations of coffee and tea consumption with incident T2D.

**TABLE 1:** Baseline characteristics of participants in the EPIC-InterAct study (*n* = 21,437)^1^

Characteristics	Subcohort (*n* = 12,477)	Incident cases (*n* = 9542)
Average years of follow-up	12.3 (11.2, 13.3)	6.8 (4.4, 9.4)
Age (y)	52.4 (9.2)	55.7 (7.6)
Sex (%men)	37.9	49.9
Physical activity level (%)		
Inactive	22.4	29.7
Moderately inactive	33.7	32.9
Moderately active	23.1	20.3
Active	20.8	17.2
Highest educational level (%)		
None	6.7	9.0
Primary school	31.9	41.4
Technical/occupational school	24.1	24.6
Secondary school	15.1	10.6
Higher education (incl. university)	21.4	13.4
Family history of diabetes (%)	17.9	35.8
Smoking status (%)		
Never	46.5	40.2
Former	27.0	31.2
Current smoker	26.1	28.1
BMI (kg/m^2^)	25.8 (4.0)	29.7 (4.8)
Energy intake (kcal/d)	2,041 (604)	2,072 (634)
Alcohol consumption (g/d)	6.5 (1.0, 18.5)	6.2 (0.7, 19.6)

1 Mean + SD or median + IQR is presented for each continuous variable. Proportions are presented for categorical variables.

**TABLE 2:** Dietary consumption levels, genetic susceptibility to metabolic traits, and their associations with incidence of type 2 diabetes: EPIC-InterAct study (*n* = 21,437)

Exposure variable	Subcohort (*n* = 12,477)	Incident cases (*n* = 9542)	Hazard ratio (95% CI)^1^	
			Model 1	Model 1 + BMI
Foods or beverages, portion size				
Fruits, 100 g/d	188 (102, 307)	179 (96, 301)	1.03 (1.00, 1.05)	1.00 (0.97, 1.04)
Green leafy vegetables, 90 g/d	11.9 (2.5, 33.4)	8.6 (1.4, 30.4)	1.18 (1.06, 1.31)	1.12 (1.00, 1.26)
Root vegetables, 80 g/d	11.8 (4.4, 28.6)	10.5 (3.8, 27.0)	0.85 (0.75, 0.96)	0.90 (0.80, 1.02)
Wholegrains, 40 g/d	42.7 (3.3, 102.5)	43.9 (2.5, 102.5)	0.99 (0.96, 1.03)	0.99 (0.96, 1.03)
Rice, 100 g/d	15.0 (5.2, 28.6)	14.4 (3.6, 26)	1.04 (0.88, 1.23)	0.75 (0.45, 1.24)
Fermented dairy, 125 mL/d	73.9 (34.7, 141.0)	66.9 (28.6, 132.1)	0.97 (0.92, 1.02)	0.96 (0.89, 1.02)
Nuts and seeds, 30 g/d	0.7 (0, 2.9)	0.3 (0, 1.7)	1.05 (0.83, 1.33)	1.07 (0.87, 1.32)
Legumes, 35 g/d^2^	6.2 (0.6, 23.1)	6.2 (0.5, 23.1)	1.09 (0.96, 1.24)	1.04 (0.94, 1.16)
Red meat, 144 g/d	37.7 (18.3, 65.5)	43.2 (22.5, 71.1)	1.70 (1.39, 2.09)	1.21 (1.00, 1.47)
Processed meat, 75 g/d	28.6 (14.9, 49.6)	32.8 (17.6, 56.2)	1.48 (1.31, 1.66)	1.23 (1.07, 1.42)
Fish, 100 g/d	5.9 (0.6, 14.4)	6.6 (0.6, 15.0)	1.23 (0.94, 1.60)	1.12 (0.84, 1.49)
Egg and egg products, 50 g/d	14.3 (6.8, 24.4)	15.2 (7, 27.1)	1.25 (1.10, 1.42)	1.03 (0.81, 1.32)
SSB, 336 mL/d	0 (0, 42.9)	0 (0, 57.1)	1.36 (1.17, 1.58)	1.18 (1.02, 1.36)
Coffee, 260 mL/d	296 (98.9, 580)	300 (95.7, 581)	0.94 (0.90, 0.98)	0.92 (0.89, 0.96)
Tea, 260 mL/d	5.0 (0, 200.0)	2.5 (0, 150.0)	0.88 (0.82, 0.93)	0.93 (0.87, 0.99)
Weighted PRS^3^				
PRS for type 2 diabetes	25.3 (24.8, 25.7)	25.6 (25.2, 26.1)	1.74 (1.66, 1.81)	1.80 (1.71, 1.90)
PRS for insulin resistance	0.33 (0.30, 0.37)	0.34 (0.30, 0.37)	1.03 (1.00, 1.06)	1.04 (1.00, 1.08)
PRS for BMI	10.6 (10.4, 10.8)	10.6 (10.4, 10.8)	1.19 (1.14, 1.24)	Not applicable

1 Evaluating 9542 cases from 21,437 participants and person-years of 148,760.7 under the case–cohort design, hazard ratios [95% confidence interval (CI)] for each dietary variable were estimated per portion size of each dietary item; for each polygenetic risk score (PRS), per SD. For dietary variables, Model 1 adjusted for age (=underlying time scale), sex, center, total energy intake, physical activity, education, smoking status, alcohol categories, all foods were mutually adjusted (except for legumes); and the second model adjusted for BMI. For PRSs, Model 1 adjusted for age (=underlying time scale), sex, center, first 5 principal components for population stratification. The BMI adjustment was not made when analyzing the PRS for BMI.

2 For legumes, the analysis evaluated 749 incident type 2 diabetes cases and 17,880 participants in total, because of no assessment of legume consumption in Denmark, where the consumption is likely to be low.

3 Weighted PRS were constructed according to published results from genome-wide association studies: SDs of weighted PRSs for type 2 diabetes were 0.70; for insulin resistance, 0.05; and for BMI, 0.26.

**FIGURE 1:**
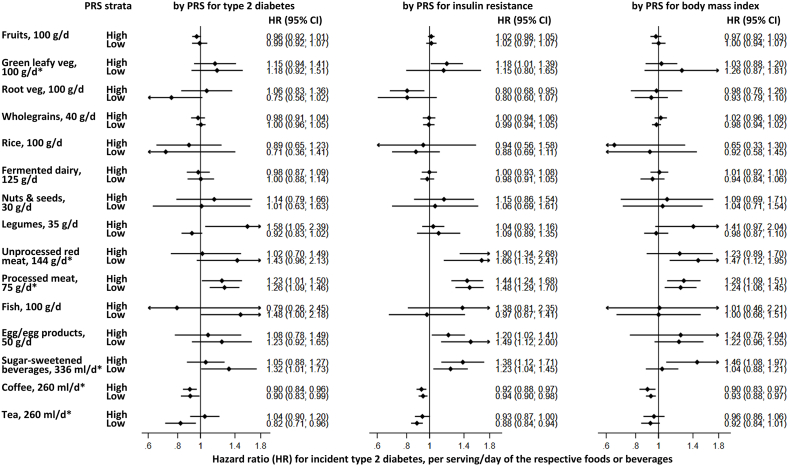
Prospective associations of food and beverage intake with incidence of type 2 diabetes by weighted polygenetic risk scores for the type 2 diabetes, insulin resistance, and higher BMI: EPIC-InterAct study (*n* = 21,437). Cox proportional hazard regression analyses were stratified by each median value of the polygenetic risk scores (PRSs) and performed to estimate hazard ratios (HRs) and 95% confidence intervals (CIs) per portion size adjusted for potential confounders (see Table 2 footnote). Individual results from interaction tests are available in Supplemental Tables 2 and 3. ∗An average association (without stratification) showed *P* < 0.05 (Table 2).

No interactions were evident for any combinations of food/beverages and PRS. Correcting for multiple tests, we found no significant evidence of multiplicative interactions of the dietary variables with each of the weighted PRSs (Figure 1, Supplemental Table 2, Supplemental Figure 2). In the most adjusted model, exp(β_interaction_) (95% confidence interval) ranged from 0.84 (0.64, 1.10) (root vegetables and PRS for T2D) to 1.45 (0.78, 2.76) (fish and PRS for T2D). No significant evidence was detected for unweighted PRSs and no evidence for additive interactions (Supplemental Tables 3 and 4). The lowest *P* value for a multiplicative interaction was 0.006 (rice and PRS for IR); and for an additive interaction, 0.0033 (rice and PRS for T2D). None passed a significance threshold determined by a Benjamini–Yekutieli procedure (the lowest *P* > 0.0003) (Supplemental Figure 2).

Post hoc analyses using 247 SNPs related to metabolic traits showed that none had significant interaction with 1 of the 15 dietary factors for incident T2D, with a Benjamini–Yekutieli correction (Supplemental Figure 3). Of 247 SNPs, 141 SNPs showed *P* <0.05 for an interaction with 1 or more of the 15 dietary factors, with or without adjustment for BMI (Supplemental Table 5). Matched to SNP databases [39], 115 SNPs were previously reported to predict nonmetabolic traits in addition to T2D- or obesity-related traits, such as smoking habits (76 SNPs), cancer (29 SNPs), and schizophrenia (24 SNPs) (Supplemental Table 6).

## Discussion

In this large prospective study of T2D incidence across 8 European countries included in EPIC-InterAct, we did not find strong evidence that the association between 15 major food groups and risk of developing T2D differed by polygenic risk of obesity, IR, or T2D on either a multiplicative or additive scale.

The associations between the 3 PRSs and T2D risk were consistent with the previous literature [[23], [24], [25]], confirming connections of adiposity and IR to the clinical manifestation of T2D. Similarly, all associations between foods or beverages and incident T2D, except for green leafy vegetables, were consistent with available summary evidence (Supplemental Table 1). In our analyses, usual green leafy vegetable intake was positively associated with T2D after adjusting for BMI, which was unexpected. The reasons are unclear but may be due to methodological differences arising from the different datasets: the current analysis included EPIC-InterAct data from all available research centers, whereas a prior analysis excluded data for some centers [40]. When we repeated our current analyses to be consistent with the previous analysis by excluding data from those centers, we replicated the previous findings.

There has been inconsistent evidence for interactions between dietary patterns and T2D genetic risk on developing T2D [18,19,41] and where they have been identified, replication is still needed [19]. Some previous studies investigated interactions with foods and beverages making up their dietary score where results concur with our null findings [18,19]. Our current findings suggest that the higher habitual consumption of a portion of red meat, processed meat, or a portion of SSB is associated with higher risk of T2D, regardless of baseline genetic risk of obesity, IR, or T2D. For example, even among individuals with a lower genetic predisposition to a higher BMI, those who habitually consumed a portion a day of red meat had 54% higher incidence of developing T2D (Figure 1). This is in contrast to findings from a study among United States men [20], where authors reported an interaction between red meat consumption and T2D PRS and the odds of T2D. A prospective analysis in UK Biobank (5663 incident cases, >357,000 participants) found interactions for processed red meat [19]. However, both these studies differed from our study in that they did not statistically control for dietary variables related to either red or processed meat consumption such as dairy intake.

Our findings also suggest that the habitual consumption of coffee or tea was inversely associated with T2D, regardless of baseline genetic risk of obesity, IR, or T2D. On the other hand, when examining individual genetic variants, a previous EPIC-InterAct analysis found a stronger T2D risk-lowering effect of higher coffee intake in those with a T allele of the *TCFL2* variant rs12255372 [10], confirmed in the current analysis (Supplemental Table 6). A genome-wide interaction analysis in UK Biobank identified 5 variant-dietary pairs reaching genome-wide significance for glycated hemoglobin [42]. Those findings were not yet replicated in independent datasets and such interaction detection could depend on hypothesis-free or -driven approaches and criteria used (e.g., α corrected for false discovery rate). Our post hoc analyses of individual SNPs indicated that gene–diet interactions, if present, were weak and nonsignificant, consistent with the multifactorial nature of T2D etiology. Future population-based studies of gene–diet interactions may benefit from incorporating more biologically informed approaches, such as evaluating SNPs grouped by shared molecular functions or pathways, rather than by their aggregate effects on T2D risk [37]. In line with this concept, we analyzed PRSs for BMI and IR that represented etiologic pathways toward T2D, but further work at more detailed molecular and functional levels will be needed to advance this field.

As study strengths, our analysis included 9542 incident cases of T2D, considerably higher than previous observational studies (*N* cases <6000, [19]). We comprehensively investigated the interaction of food and beverage consumption with 3 separate metabolic PRSs on T2D incidence, thus testing key pathways of obesity and IR for development of T2D. We used a systematic approach in selecting and evaluating foods and beverages, accounted for multiple testing to reduce the chance of false positive findings, tested both multiplicative and additive food-gene interactions.

Our study has some limitations. Dietary intakes and study covariates, including anthropometric measures and physical activity, were derived from self-reported data collected only at baseline, which are subject to measurement errors. Such error may have introduced bias in quantitative estimations, affected the detection of gene–diet interactions, and potentially explained our null findings. This study included the largest number of incident cases of T2D to date to address the research question; however, it is possible that the study was still not sufficiently large to detect true interactions as statistically significant after adjustment for multiple testing. Additionally, the genetic variants analyzed in this study were identified based on statistical associations with complex phenotypes rather than known mechanistic pathways. As a result, the lack of clear biological links to nutrient metabolism further limits the mechanistic interpretation of observed (absent) gene–diet interactions. Although the EPIC-InterAct assures broad generalizability across European populations because of its large scale and participants from multiple countries, the extent to which our findings can be extrapolated to non-European populations remains uncertain. In this regard, our findings were consistent with those from a study in China that recruited 550,000 Chinese participants and evaluated a healthy-lifestyle score based on high consumption of vegetables, fruits, and wholegrains and low consumption of meat and 3 PRSs (T2D, IR, and β-cell dysfunction) [22]. Our research focused on investigating the interplay between dietary factors and genetic predisposition with respect to T2D etiology, prioritizing food groups known to be associated with T2D. Therefore, we cannot rule out potential interactions with other foods or beverages, dietary patterns or other genetic loci that were not tested. There may also be factors not investigated by us that may contribute to the variation in pathways to metabolic disease. For instance, Berry et al. [43] reported high interindividual variability in postprandial glucose to the same meal with influences from macronutrient composition, gut microbiome, meal context, and genetics. This places the contribution of genetics into perspective alongside other factors relevant in evaluating the feasibility for personalized nutrition for glycemic control, a determinant of T2D.

Findings from the prospective EPIC-InterAct study in 8 European countries suggest that the associations of 15 food groups and beverages with T2D incidence are not modified by an individual’s genetic predisposition for higher BMI, IR, or T2D. Although research into personalized nutrition approaches should continue, our findings underscore the current methodological challenges in identifying gene–diet interactions using genetic risk scores. At the same time, they provide consistent support for the effectiveness of population-wide, food-based dietary guidance in preventing T2D.

## Author contributions

The authors’ responsibilities were as follows – SXL, FI, NGF, NJW: had full access to all of the data in the study and take responsibility for the integrity of the data and the accuracy of the data analysis; SXL, FI: performed the statistical analyses; SXL: wrote the first draft of the manuscript; NJW, CL, NGF, SJS: coordinated the EPIC-InterAct project, with NJW as chief investigator; NGF, NJW, the Working Group initially, and then all authors: contributed to interpretation of data, revised the article critically for important intellectual content, and approved the final version of the manuscript. The corresponding author attests that all listed authors meet authorship criteria and that no others meeting the criteria have been omitted; NGF, NJW: are the guarantors of this work and, as such, had full access to all the data in the study and take responsibility for the integrity of the data and the accuracy of the data analysis; and all authors: read and approved the final manuscript.

## Copyright/license

The corresponding author has the right to grant on behalf of all authors and does grant on behalf of all authors, a worldwide license to the Publishers and its licensees in perpetuity, in all forms, formats, and media (whether known now or created in the future), to *1*) publish, reproduce, distribute, display, and store the Contribution, *2*) translate the Contribution into other languages, create adaptations, reprints, include within collections and create summaries, extracts, and/or abstracts of the Contribution, *3*) create any other derivative work(s) based on the Contribution, *4*) to exploit all subsidiary rights in the Contribution, *5*) the inclusion of electronic links from the Contribution to third-party material wherever it may be located, and *6*) license any third party to do any or all of the above.

## Transparency declaration

The lead authors and the corresponding author confirm that this manuscript is an honest, accurate, and transparent account of the study being reported. No key aspects of the study have been omitted and any discrepancies from the study as originally planned have been explained.

## Data availability

EPIC-InterAct study data cannot be deposited publicly because these collaborative data originate from multiple research institutions across 8 European countries with different legal frameworks. The authors confirm that researchers seeking the analysis dataset for this work can submit a data request to the EPIC study steering committee via the Cambridge team by emailing nick.wareham@mrc-epid.cam.ac.uk.

## Funding

The EPIC-InterAct project is funded by the EU FP6 programme LSHM_CT_2006_037197). SXL acknowledge the Commonwealth Scholarship Commission and the Cambridge Trust. SXL, NGF, FI, CL, RS, and NJW acknowledge Medical Research Council Epidemiology Unit core support from MC_UU_00006/1 and MC_UU_00006/3. NGF and NJW also acknowledge National Institute for Health and Care Research (NIHR) Biomedical Research Centre Cambridge: Nutrition, Diet, and Lifestyle Research Theme (IS-BRC-1215-20014) and NGF is a NIHR Senior Investigator. The views expressed are those of the authors and not necessarily those of the NIHR or the Department of Health and Social Care. MBS was supported by a grant from the German Federal Ministry of Education and Research (BMBF) to the German Center for Diabetes Research (DZD) and the State of Brandenburg (82DZD03D03). JSZ received funding from Westlake University (No. YSYY0209) and the European Union’s Horizon 2020 research and innovation program under the Marie Sklodowska-Curie grant agreement No 701708. PA, EA, and MDC were supported by the Health Research Fund of the Spanish Ministry of Health. PA was supported by Basque Country Government. EA was supported by Navarre Regional Government. MDC is supported by Murcia Regional Government (No 6236). PWF was supported by the Swedish Research Council, Novo Nordisk, Swedish Diabetes Association, Swedish Heart-Lung Foundation, and European Research Council. PJ was supported by The Health Research Funds (RD12/0036/0018) and AGAUR, Generalitat de Catalunya (exp. 2014 SGR 726). VAK was supported by the German Cancer Aid and BMBF. KO and AT would like to acknowledge the Danish Cancer Society. SP would like to acknowledge Compagnia di San Paolo. OR was supported by the Västerbotten County Council. EPIC Bilthoven and Utrecht acknowledge the Dutch Ministry of Public Health, Welfare and Sports (VWS), Netherlands Cancer Registry (NKR), LK Research Funds, Dutch Prevention Funds, Dutch Zorg Onderzoek Nederland, and World Cancer Research Fund. EPIC-Oxford was supported by Cancer Research UK (C8221/A29017) and UK Medical Research Council (MR/M012190/1). TYNT was supported by a Nuffield Department of Population Health Intermediate Fellowship. ER was supported by the Imperial College Biomedical Research Centre.

## Conflict of interest

The authors report no conflicts of interest.

## References

[1] Ong K.L., Stafford L.K., McLaughlin S.A., Boyko E.J., Vollset S.E., Smith A.E. (2023). Global, regional, and national burden of diabetes from 1990 to 2021, with projections of prevalence to 2050: a systematic analysis for the Global Burden of Disease Study 2021. Lancet..

[2] Forouhi N.G., Misra A., Mohan V., Taylor R., Yancy W. (2018). Dietary and nutritional approaches for prevention and management of type 2 diabetes. BMJ.

[3] Ley S.H., Hamdy O., Mohan V., Hu F.B. (2014). Prevention and management of type 2 diabetes: dietary components and nutritional strategies. Lancet..

[4] Dyson P.A., Twenefour D., Breen C., Duncan A., Elvin E., Goff L. (2018). Diabetes UK evidence-based nutrition guidelines for the prevention and management of diabetes, Diabet. Med..

[5] ADA (2017). Standards of Medical Care in Diabetes – 2017. Diabetes Care.

[6] Collins F.S., Varmus H. (2015). A new initiative on precision medicine. N. Engl. J. Med..

[7] Wang D.D., Hu F.B. (2018). Precision nutrition for prevention and management of type 2 diabetes. Lancet Diabetes Endocrinol.

[8] Wirström T., Hilding A., Gu H.F., Östenson C.-G., Björklund A. (2013). Consumption of whole grain reduces risk of deteriorating glucose tolerance, including progression to prediabetes. Am. J. Clin. Nutr..

[9] Fisher E., Boeing H., Fritsche A., Doering F., Joost H.-G., Schulze M.B. (2009). Whole-grain consumption and transcription factor-7-like 2 (TCF7L2) rs7903146: gene-diet interaction in modulating type 2 diabetes risk. Br. J. Nutr..

[bib10] The InterAct Consortium (2016). Investigation of gene–diet interactions in the incretin system and risk of type 2 diabetes: the EPIC-InterAct study. Diabetologia.

[11] Dudbridge F. (2013). Power and predictive accuracy of polygenic risk scores. PLOS Genet..

[12] Li S.X., Imamura F., Schulze M.B., Zheng J., Ye Z., Agudo A. (2018). Interplay between genetic predisposition, macronutrient intake and type 2 diabetes incidence: analysis within EPIC-InterAct across eight European countries. Diabetologia.

[13] Merino J., Guasch-Ferré M., Ellervik C., Dashti H.S., Sharp S.J., Wu P. (2019). Quality of dietary fat and genetic risk of type 2 diabetes: individual participant data meta-analysis. BMJ.

[14] Qi Q., Chu A.Y., Kang J.H., Jensen M.K., Curhan G.C., Pasquale L.R. (2012). Sugar-sweetened beverages and genetic risk of obesity. N. Engl. J. Med..

[15] Brunkwall L., Chen Y., Hindy G., Rukh G., Ericson U., Barroso I. (2016). Sugar-sweetened beverage consumption and genetic predisposition to obesity in 2 Swedish cohorts. Am. J. Clin. Nutr..

[16] Olsen N.J., Ängquist L., Larsen S.C., Linneberg A., Skaaby T., Husemoen L.L.N. (2016). Interactions between genetic variants associated with adiposity traits and soft drinks in relation to longitudinal changes in body weight and waist circumference. Am. J. Clin. Nutr..

[17] Jia X., Xuan L., Dai H., Zhu W., Deng C., Wang T. (2021). Fruit intake, genetic risk and type 2 diabetes: a population-based gene–diet interaction analysis. Eur. J. Nutr..

[18] Ericson U., Hindy G., Drake I., Schulz C.-A., Brunkwall L., Hellstrand S. (2018). Dietary and genetic risk scores and incidence of type 2 diabetes. Genes Nutr.

[19] Zhuang P., Liu X., Li Y., Wan X., Wu Y., Wu F. (2021). Effect of diet quality and genetic predisposition on hemoglobin A1c and type 2 diabetes risk: gene-diet interaction analysis of 357,419 individuals. Diab. Care.

[20] Qi L., Cornelis M.C., Zhang C., van Dam R.M., Hu F.B. (2009). Genetic predisposition, Western dietary pattern, and the risk of type 2 diabetes in men. Am. J. Clin. Nutr..

[21] Dietrich S., Jacobs S., Zheng J., Meidtner K., Schwingshackl L., Schulze M.B. (2019). Gene-lifestyle interaction on risk of type 2 diabetes: a systematic review. Obes. Rev..

[22] Li H., Khor C.-C., Fan J., Lv J., Yu C., Guo Y. (2020). Genetic risk, adherence to a healthy lifestyle, and type 2 diabetes risk among 550,000 Chinese adults: results from 2 independent Asian cohorts. Am. J. Clin. Nutr..

[23] Yengo L., Sidorenko J., Kemper K.E., Zheng Z., Wood A.R., Weedon M.N. (2018). Meta-analysis of genome-wide association studies for height and body mass index in ∼700000 individuals of European ancestry. Hum. Mol. Genet..

[24] Lotta L.A., Gulati P., Day F.R., Payne F., Ongen H., van de Bunt M. (2017). Integrative genomic analysis implicates limited peripheral adipose storage capacity in the pathogenesis of human insulin resistance. Nat. Genet..

[25] Mahajan A., Taliun D., Thurner M., Robertson N.R., Torres J.M., Rayner N.W. (2018). Fine-mapping type 2 diabetes loci to single-variant resolution using high-density imputation and islet-specific epigenome maps. Nat. Genet..

[26] InterAct Consortium The, Langenberg C., Sharp S., Forouhi N.G., Franks P.W., Schulze M.B. (2011). Design and cohort description of the InterAct Project: an examination of the interaction of genetic and lifestyle factors on the incidence of type 2 diabetes in the EPIC Study. Diabetologia.

[27] Locke A.E., Kahali B., Berndt S.I., Justice A.E., Pers T.H., Day F.R. (2015). Genetic studies of body mass index yield new insights for obesity biology. Nature.

[28] Morris A.P., Voight B.F., Teslovich T.M., Ferreira T., Segrè A.V., Steinthorsdottir V. (2012). Large-scale association analysis provides insights into the genetic architecture and pathophysiology of type 2 diabetes. Nat. Genet..

[29] Choi S.W., Mak T.S.-H., O’Reilly P.F. (2020). Tutorial: a guide to performing polygenic risk score analyses. Nat. Protoc..

[30] Schwingshackl L., Hoffmann G., Lampousi A.-M., Knüppel S., Iqbal K., Schwedhelm C. (2017). Food groups and risk of type 2 diabetes mellitus: a systematic review and meta-analysis of prospective studies. Eur. J. Epidemiol..

[31] Neuenschwander M., Ballon A., Weber K.S., Norat T., Aune D., Schwingshackl L. (2019). Role of diet in type 2 diabetes incidence: umbrella review of meta-analyses of prospective observational studies. BMJ.

[32] Kroke A., Klipstein-Grobusch K., Voss S., Möseneder J., Thielecke F., Noack R. (1999). Validation of a self-administered food-frequency questionnaire administered in the European Prospective Investigation into Cancer and Nutrition (EPIC) study: comparison of energy, protein, and macronutrient intakes estimated with the doubly labeled water, urinary nitrogen, and repeated 24-h dietary recall methods. Am. J. Clin. Nutr..

[33] Bingham S., Day N. (1997). Using biochemical markers to assess the validity of prospective dietary assessment methods and the effect of energy adjustment. Am. J. Clin. Nutr..

[34] Lassen A.D., Christensen L.M., Fagt S., Trolle E. (2020).

[35] Agency F.S., Mills A., Patel S., Crawley H. (2002).

[36] Vanderweele T. (2015).

[37] Aschard H. (2016). A perspective on interaction effects in genetic association studies. Genet. Epidemiol..

[38] Benjamini Y., Yekutieli D. (2001). The control of the false discovery rate in multiple testing under dependency. Ann. Stat..

[39] Oscanoa J., Sivapalan L., Gadaleta E., Dayem Ullah A.Z., Lemoine N.R., Chelala C. (2020). SNPnexus: a web server for functional annotation of human genome sequence variation (2020 update). Nucleic Acids Res.

[40] Cooper A.J., Forouhi N.G., Ye Z., Buijsse B., Arriola L., Balkau B. (2012). Fruit and vegetable intake and type 2 diabetes: EPIC-InterAct prospective study and meta-analysis. Eur. J. Clin. Nutr..

[41] Langenberg C., Sharp S.J., Franks P.W., Scott R.A., Deloukas P., Forouhi N.G. (2014). Gene-lifestyle interaction and type 2 diabetes: the EPIC interact case-cohort study. PLOS Med.

[42] Westerman K.E., Miao J., Chasman D.I., Florez J.C., Chen H., Manning A.K. (2021). Genome-wide gene–diet interaction analysis in the UK Biobank identifies novel effects on hemoglobin A1c. Hum. Mol. Genet..

[43] Berry S.E., Valdes A.M., Drew D.A., Asnicar F., Mazidi M., Wolf J. (2020). Human postprandial responses to food and potential for precision nutrition. Nat. Med..

