# Supplementary material for: Interactions between genetic predisposition to obesity, insulin resistance and type 2 diabetes risk, and food or beverage intake for incident type 2 diabetes: European Prospective Investigation into Cancer and Nutrition (EPIC) InterAct case–cohort study
Source: Am J Clin Nutr. 2026 Jan 16;123(3):101198. doi: 10.1016/j.ajcnut.2026.101198 (PMC12975352; doi:10.1016/j.ajcnut.2026.101198)
Supplement: multimedia component 1 [file mmc1.docx]

**Supplemental materials of Sherly X Li *et al*., “Interactions between genetic predisposition to obesity, insulin resistance and type 2 diabetes risk and food or beverage intake for incident type 2 diabetes: European Prospective Investigation into Cancer (EPIC) InterAct case-cohort study”**

Contents

[Supplemental Text. 2](#_Toc212797780)

[DNA extraction and genotyping 2](#_Toc212797781)

[Descriptions of the covariates 2](#_Toc212797782)

[Longitudinal associations of single foods/beverages and polygenic risk scores (PRSs) with incident type 2 diabetes 3](#_Toc212797783)

[Supplemental Figure 1. EPIC-InterAct case-cohort design and the number of participants in this analysis. 4](#_Toc212797784)

[Supplemental Table 1. Definition of foods and beverages included in the analyses: EPIC-InterAct study 5](#_Toc212797785)

[Supplemental Figure 2. Q-Q plots of results from tests for multiplicative and additive interactions between fifteen dietary factors and three polygenic risk scores. 5](#_Toc212797786)

[Supplemental Table 2. Multiplicative interaction between three weighted polygenic risk scores (wPRSs) and food or beverage intakes on incident type 2 diabetes: EPIC-InterAct study^1^ 6](#_Toc212797787)

[Supplemental Table 3. Multiplicative interaction between three unweighted polygenic risk scores (uwPRSs) and food or beverage intakes on incident type 2 diabetes: EPIC-InterAct study.^1^ 8](#_Toc212797788)

[Supplemental Table 4. Additive interaction between three weighted polygenic risk scores (wPRSs) and food or beverage intakes on incident type 2 diabetes: EPIC-InterAct study.^1^ 10](#_Toc212797789)

[Supplemental Figure 3. Q-Q plots of results from tests for multiplicative interactions between 15 food items and 247 single nucleotide polymorphisms contributing to the polygenic risk scores: EPIC-InterAct case-cohort analysis. 11](#_Toc212797790)

[Supplemental Table 5. Single nucleotide polymorphisms (SNPs) with a potential interaction (crude p<0.05) with one or more of 15 food items for incidence of type 2 diabetes: EPIC-InterAct case-cohort analysis^1^. 13](#_Toc212797791)

[Supplemental Table 6. Genetic association results of the single nucleotide polymorphisms (SNPs) identified to have a potential interaction with one or more of fifteen food groups for incident type 2 diabetes in EPIC-InterAct case-cohort study. (](#_Toc212797792)Supplemental Excel file)

[References 21](#_Toc212797793)

# Supplemental Text.

DNA extraction and genotyping: Samples of ascertained cases and subcohort participants were retrieved from liquid nitrogen storage in the International Agency for Research on Cancer (n=20,794) or local biobanks (n=9,247) in parallel with ongoing case verification efforts (1). DNA was extracted from up to 1ml of buffy coat for each individual from a citrated blood sample. Standard procedures on an automated Autopure LS® DNA extraction system (Qiagen, Germany) with PUREGENE® chemistry (Qiagen, Germany) were used and the DNA was hydrated overnight prior to further processing. Non-DNA samples (one 500µl serum, two 500µl plasma, and one 500µl erythrocyte straw(s)) and DNA samples were shipped to the central holding bay in Cambridge, where they are stored until dispatch for genotyping or biomarker measurement. Delivery of all samples was completed in July 2010. All samples are logged and stored in a Human Tissue Authority licensed facility. DNA samples were quantified by Picogreen and normalised to 50 ng/µl at the MRC Epidemiology Unit laboratory before aliquots were sent to the Wellcome Trust Sanger Institute (WTSI) in bar-coded 96-well plates. Upon receipt and electronic loading of sample information (anonymised sample identifier, gender, broad geographical region, and sample concentration) all samples were checked on agarose gels for degradation. Only non-degraded samples with sufficient DNA entered the genotyping pipeline.

Descriptions of the covariates: Baseline, standardised questionnaires and measurements were used across participating EPIC study centres to collect non-dietary covariates. The following covariates were considered in this study: physical activity, education status, smoking status, alcohol consumption, body weight and height to calculate body-mass index (weight divided by squared height, kg/m^2^), and family history of diabetes.

Physical activity was assessed via a brief self-report questionnaire covering both occupational and recreational activity (2). An overall categorical physical activity index was derived, with four levels: inactive, moderately inactive, moderately active, and active. Highest level of education attained was self-reported. Categories of education status were standardized across the participating study centres (3): none (or no formal schooling), primary, technical or vocational school, secondary school, further education or university degree. Smoking history was self-reported. Participants in this current study were then categorised into never smokers, former smokers, and current smokers. Alcohol intake at baseline was self-reported, standardized to be expressed of the unit of grams per day in this study, and six categories by alcohol consumption were modelled categorically in our regression analysis. Weight and height were measured using standardised protocols (without shoes, light clothing) in most centres; in some centres (e.g. Oxford) self-reported values were used. Body mass index (BMI) was calculated as weight (kg) divided by height (m)^2^. Family history was collected via questionnaire at baseline, asking whether a first-degree family member (parents) had been diagnosed with type 2 diabetes. This covariate was recorded dichotomously (yes/no).

In addition to those covariates, study centers, total energy intake, and five genetic principal components were included as covariates in our regression analysis evaluating gene-diet interactions. Total energy intake was estimated with self-reported food and beverage consumption and local food composition table(s) as described in the method publications of the European Prospective Investigation into Cancer and Nutrition study cohort (EPIC). Five genetic principal components were derived previously from principal component analysis of genome-wide association results using QUICKTEST version 0.98 and used as covariates to control for population stratification.

Longitudinal associations of single foods/beverages and polygenic risk scores (PRSs) with incident type 2 diabetes. We examined an association of each of the fifteen dietary items with incident T2D with Prentice-weighted Cox regression models stratified by country. The model adjusted for age as the underlying timescale, sex, centers, total energy intake, physical activity (inactive, moderately inactive, moderately active, active), education (none, primary school, technical/professional, secondary school, longer education, including university), smoking (never, former, current smoker), alcohol consumption, and fifteen foods and beverages (mutually adjusted); legume variable in Denmark was not included in the model. Country-specific estimates were pooled using random-effects meta-analysis. Heterogeneity across the countries was quantified with I^2^.

We evaluated an association of each of the three PRSs with incident T2D, modelling Prentice-weighted Cox regression stratified by country and genotyping chip. The model adjusted for age (the underlying timescale), sex, center, five principal components of genome-wide association results to account for population stratification, and BMI, except where the BMI PRS was of focus. Country- and gene-chip-specific estimates of HR were combined using random-effects meta-analysis. Heterogeneity across the countries was quantified with I^2^.

*

Supplemental Figure 1. EPIC-InterAct case-cohort design and the number of participants in this analysis. Final sample of 21,437 participants included in the current analysis, which includes 9,542 cases (8,960 + 582) and 11,895 non-cases from the subcohort. *Sequentially, 5,287 participants without genetic data were excluded, 592 without dietary data were excluded, and then 463 without covariate data were excluded for the current study.

| Supplemental Table 1. Definition of foods and beverages included in the analyses: EPIC-InterAct study  \| **Foods and beverages included (reference to published meta-analysis)** \| **Definition and examples** \| \| --- \| --- \| \| Fruits(4) \| apples, oranges, grape, stone fruits, mixed fruits (dried fruits, fruit salad) \| \| Green leafy vegetables(4) \| lettuce, spinach, swiss chard leaf \| \| Root vegetables(4) \| carrot, celeriac, radish, parsnip, beetroot. Exclude potato. \| \| Wholegrains and non-white breads (5) \| bread and crispbreads (non-white), grains (e.g. couscous, semolina, polenta, pearl barley) \| \| Rice(6) \| white and brown rice \| \| Legumes(7) \| red kidney beans, chickpeas, lentils \| \| Nuts and seeds(7) \| nuts and seeds, tree nuts, peanuts, chestnuts \| \| Fermented dairy(8,9) \| yoghurt and fermented milk (e.g. kefir), cheese (e.g. ricotta, cheddar) \| \| Unprocessed red meat(10) \| beef, lamb, pork, goat \| \| Processed meat(10) \| bacon, ham, cold meat \| \| Fish(11) \| fish including oily and white fish but not including crustaceans/fish products \| \| Egg and egg products(12) \| egg, pickled egg, egg powder \| \| Sugar Sweetened Beverages (SSB)(13) \| carbonated, soft, isotonic drinks, diluted syrups (e.g. cola, lemonade, sweetened or sugar reduced). Based on the proxy: total soft drinks minus artificially sweetened beverages \| \| Coffee(14) \| caffeinated, decaffeinated and partially caffeinated \| \| Tea(15) \| black and green teas \| |  |  |
| --- | --- | --- | --- | --- | --- | --- | --- | --- | --- | --- | --- | --- | --- | --- | --- | --- | --- | --- | --- | --- | --- | --- | --- | --- | --- | --- | --- | --- | --- | --- | --- | --- | --- | --- |


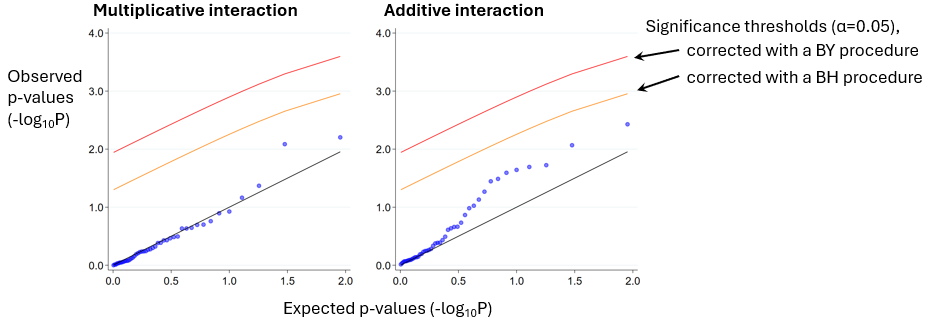


Supplemental Figure 2. Q-Q plots of results from tests for multiplicative and additive interactions between fifteen dietary factors and three polygenic risk scores. Country- and gene-chip specific prentice-weighted multivariable-adjusted Cox regression model was fitted (N=21,437; 9,542 cases and 11,895 non-cases), followed by random-effects meta-analysis, separately to obtain a parameter estimate for each pair of the dietary factor and the polygenic risk score. Results from (Left) a multiplicative interaction and (Right) an additive interaction are presented as a Q-Q plot, derived from 15×3 parameter estimates, as a set of multiple tests separately for the two types of interaction. The red line represents significant thresholds across the ranks of p-values according to a Benjamini–Yekutieli (BY) correction; and the orange line, a Benjamini- Hochberg (BH) correction.

# Supplemental Table 2. Multiplicative interaction between three weighted polygenic risk scores (wPRSs) and food or beverage intakes on incident type 2 diabetes: EPIC-InterAct study^1^

| **Food or beverage,  portion size** | **Model** | **Relative difference in HRs (HR_1_/HR_0_)**^2^ **as a measure of multiplicative interaction** | | | | | | | | |
| --- | --- | --- | --- | --- | --- | --- | --- | --- | --- | --- |
|  |  | **wPRS for type 2 diabetes** | | | **wPRS for insulin resistance** | | | **wPRS for body mass index**^3^ | | |
|  |  | **HR_1_/HR_0_ (95% CI)** | ***P*** | **I^2^, %** | **HR_1_/HR_0_ (95% CI)** | ***P*** | **I^2^, %** | **HR_1_/HR_0_ (95% CI)** | ***P*** | **I^2^, %** |
| Fruit, 100 g/d | *1* | 0.99 (0.96, 1.02) | 0.59 | 37.0 | 1.02 (1.00, 1.05) | 0.06 | 11.4 | 1.00 (0.98, 1.02) | 0.91 | 0.0 |
|  | *2* | 0.99 (0.96, 1.02) | 0.47 | 30.2 | 1.02 (1.00, 1.04) | 0.08 | 0.0 | 1.00 (0.98, 1.02) | 0.85 | 0.0 |
|  | *3* | 0.99 (0.97, 1.02) | 0.65 | 5.0 | 1.02 (0.98, 1.05) | 0.33 | 26.9 | ‡ |  |  |
| Green leafy vegetable,  90 g/d | *1* | 0.92 (0.77, 1.10) | 0.37 | 43.2 | 1.08 (0.95, 1.22) | 0.25 | 13.4 | 1.01 (0.92, 1.10) | 0.87 | 0.0 |
|  | *2* | 0.88 (0.73, 1.06) | 0.19 | 37.6 | 1.09 (0.97, 1.23) | 0.13 | 7.0 | 1.01 (0.92, 1.11) | 0.87 | 0.0 |
|  | *3* | 0.95 (0.80, 1.15) | 0.62 | 28.8 | 1.20 (0.95, 1.50) | 0.12 | 43.5 |  |  |  |
| Root vegetable, 80 g/d | *1* | 1.01 (0.88, 1.16) | 0.90 | 18.3 | 1.02 (0.91, 1.15) | 0.72 | 16.3 | 1.00 (0.91, 1.10) | 0.96 | 0.0 |
|  | *2* | 0.98 (0.83, 1.15) | 0.80 | 24.1 | 1.03 (0.94, 1.14) | 0.51 | 0.0 | 1.00 (0.91, 1.10) | 1.00 | 0.0 |
|  | *3* | 0.84 (0.64, 1.10) | 0.20 | 60.7 | 1.02 (0.86, 1.20) | 0.84 | 30.3 |  |  |  |
| Wholegrains, 40 g/d | *1* | 0.99 (0.96, 1.02) | 0.58 | 29.4 | 1.01 (0.98, 1.03) | 0.68 | 25.2 | 1.01 (0.98, 1.04) | 0.50 | 31.6 |
|  | *2* | 0.99 (0.96, 1.03) | 0.74 | 40.6 | 1.00 (0.97, 1.03) | 0.94 | 20.4 | 1.00 (0.96, 1.04) | 0.97 | 43.8 |
|  | *3* | 1.00 (0.95, 1.06) | 0.90 | 63.8 | 0.99 (0.96, 1.02) | 0.58 | 15.8 |  |  |  |
| Rice, 100 g/d | *1* | 0.92 (0.74, 1.15) | 0.46 | 33.9 | 1.11 (0.95, 1.29) | 0.21 | 0.0 | 0.97 (0.81, 1.17) | 0.78 | 25.9 |
|  | *2* | 0.88 (0.64, 1.20) | 0.41 | 58.6 | 1.18 (1.00, 1.39) | 0.06 | 0.0 | 0.95 (0.81, 1.12) | 0.58 | 3.9 |
|  | *3* | 1.01 (0.76, 1.36) | 0.93 | 43.1 | 1.30 (1.08, 1.57) | 0.006 | 0.0 |  |  |  |
| Legumes, 35 g/d^4^ | *1* | 1.03 (0.97, 1.09) | 0.39 | 20.8 | 1.00 (0.96, 1.05) | 0.96 | 0.0 | 1.01 (0.96, 1.05) | 0.82 | 0.0 |
|  | *2* | 1.04 (0.97, 1.10) | 0.25 | 16.9 | 1.00 (0.95, 1.04) | 0.86 | 0.0 | 1.01 (0.96, 1.05) | 0.81 | 0.0 |
|  | *3* | 1.02 (0.91, 1.13) | 0.74 | 60.1 | 0.97 (0.90, 1.04) | 0.37 | 22.8 |  |  |  |
| Nuts and seeds, 30 g/d | *1* | 1.13 (0.85, 1.50) | 0.39 | 54.2 | 1.06 (0.87, 1.29) | 0.55 | 11.1 | 0.98 (0.81, 1.18) | 0.80 | 10.7 |
|  | *2* | 1.02 (0.74, 1.40) | 0.90 | 54.8 | 1.10 (0.83, 1.45) | 0.50 | 36.4 | 0.93 (0.74, 1.16) | 0.50 | 20.0 |
|  | *3* | 0.89 (0.60, 1.31) | 0.54 | 52.4 | 1.01 (0.72, 1.41) | 0.97 | 42.4 |  |  |  |
| Fermented dairy,  125 ml/d | *1* | 0.99 (0.92, 1.07) | 0.85 | 14.2 | 1.02 (0.96, 1.09) | 0.54 | 0.0 | 0.94 (0.89, 0.99) | 0.03 | 0.0 |
|  | *2* | 0.95 (0.86, 1.06) | 0.40 | 34.4 | 1.01 (0.93, 1.10) | 0.80 | 9.2 | 0.94 (0.88, 1.00) | 0.04 | 0.0 |
|  | *3* | 0.97 (0.87, 1.08) | 0.57 | 28.4 | 0.98 (0.83, 1.14) | 0.77 | 56.3 |  |  |  |
| Red meat, 144 g/d | *1* | 0.89 (0.77, 1.03) | 0.11 | 0.0 | 1.15 (1.01, 1.32) | 0.030 | 0.0 | 0.97 (0.85, 1.10) | 0.63 | 0.0 |
|  | *2* | 0.92 (0.79, 1.08) | 0.33 | 4.7 | 1.18 (1.02, 1.36) | 0.022 | 0.0 | 0.97 (0.84, 1.12) | 0.70 | 0.0 |
|  | *3* | 0.93 (0.75, 1.14) | 0.48 | 23.8 | 1.23 (1.06, 1.44) | 0.008 | 0.0 |  |  |  |
| Processed red meat,  75 g/d | *1* | 0.96 (0.84, 1.11) | 0.60 | 52.9 | 1.03 (0.95, 1.12) | 0.43 | 6.6 | 1.02 (0.94, 1.10) | 0.65 | 0.0 |
|  | *2* | 0.96 (0.81, 1.12) | 0.58 | 58.9 | 1.02 (0.91, 1.15) | 0.71 | 38.8 | 1.02 (0.94, 1.11) | 0.59 | 0.0 |
|  | *3* | 0.91 (0.77, 1.09) | 0.32 | 55.5 | 1.01 (0.86, 1.20) | 0.89 | 57.8 |  |  |  |
| Fish, 100 g/d | *1* | 1.22 (0.79, 1.89) | 0.37 | 44.0 | 1.19 (0.94, 1.51) | 0.16 | 0.0 | 0.85 (0.62, 1.16) | 0.30 | 23.4 |
|  | *2* | 1.31 (0.76, 2.26) | 0.33 | 55.4 | 1.15 (0.88, 1.49) | 0.30 | 0.0 | 0.90 (0.64, 1.26) | 0.53 | 22.4 |
|  | *3* | 1.47 (0.78, 2.76) | 0.23 | 59.2 | 1.14 (0.85, 1.53) | 0.37 | 3.4 |  |  |  |
| Egg and egg products,  50 g/d | *1* | 0.99 (0.86, 1.14) | 0.86 | 34.3 | 1.13 (0.97, 1.32) | 0.11 | 46.9 | 1.02 (0.93, 1.12) | 0.68 | 0.0 |
|  | *2* | 1.01 (0.84, 1.22) | 0.90 | 49.6 | 1.11 (0.95, 1.30) | 0.20 | 37.4 | 1.05 (0.95, 1.16) | 0.34 | 0.0 |
|  | *3* | 1.02 (0.83, 1.27) | 0.84 | 52.4 | 1.10 (0.87, 1.38) | 0.41 | 61.8 |  |  |  |
| Sugar-sweetened beverages, 366 ml/d | *1* | 1.17 (1.02, 1.34) | 0.03 | 32.0 | 0.96 (0.88, 1.05) | 0.38 | 0.0 | 0.98 (0.87, 1.12) | 0.80 | 39.0 |
|  | *2* | 1.16 (1.02, 1.33) | 0.03 | 23.1 | 0.97 (0.88, 1.07) | 0.53 | 0.0 | 0.99 (0.86, 1.14) | 0.93 | 37.9 |
|  | *3* | 1.22 (0.98, 1.52) | 0.07 | 55.5 | 0.92 (0.82, 1.04) | 0.17 | 0.0 |  |  |  |
| Coffee, 260 ml/d | *1* | 0.98 (0.94, 1.02) | 0.27 | 28.9 | 1.02 (1.00, 1.05) | 0.10 | 0.0 | 1.02 (0.99, 1.05) | 0.27 | 10.7 |
|  | *2* | 0.97 (0.93, 1.01) | 0.15 | 29.2 | 1.02 (0.99, 1.05) | 0.17 | 0.0 | 1.03 (0.99, 1.06) | 0.13 | 8.7 |
|  | *3* | 0.97 (0.93, 1.02) | 0.20 | 18.7 | 1.02 (0.99, 1.05) | 0.23 | 0.0 |  |  |  |
| Tea, 260 ml/d | *1* | 1.01 (0.98, 1.05) | 0.49 | 0.0 | 0.97 (0.94, 1.00) | 0.06 | 0.0 | 1.00 (0.96, 1.03) | 0.78 | 0.0 |
|  | *2* | 1.01 (0.97, 1.05) | 0.66 | 0.0 | 0.97 (0.93, 1.01) | 0.10 | 0.0 | 0.98 (0.94, 1.01) | 0.23 | 0.0 |
|  | *3* | 1.00 (0.93, 1.06) | 0.90 | 20.3 | 0.98 (0.92, 1.03) | 0.41 | 14.7 |  |  |  |

^1^ N cases=9,542 incident T2D cases, N sample=21,437.

^2^ A beta coefficient (β) for the interaction between each weighted PRS and each of the foods and beverages on incident T2D was estimated per one unit difference for each PRS and food/beverage. One unit of PRS was 1 standard deviation in the EPIC InterAct subgroup, and one unit of foods/beverages was a standard portion size for each, as written on the first column. Thus, exp(β), for example, represented a ratio of two hazard ratios (HR_1_/HR_0_) consisting of HR_1_ per 1 portion of food/beverage when PRS=+1 SD and HR_0_ per 1 portion of food/beverage when PRS=0. Estimates were adjusted for age (=underlying time scale), sex, centre, total energy intake, first 5 principal components for population stratification (Model 1); model 1 + physical activity, education, smoking, sex-specific alcohol categories, and other food/beverage consumption listed (Model 2); and model 2+ baseline BMI (Model 3). As exception, legume consumption was not evaluated and not adjusted for in Denmark. §
^3^ The analyses using the BMI PRS did not adjust for baseline BMI.

^4^ N=7,749 incident T2D cases/17,880 total participants, as legume consumption was not assessed in Denmark.

# Supplemental Table 3. Multiplicative interaction between three unweighted polygenic risk scores (uwPRSs) and food or beverage intakes on incident type 2 diabetes: EPIC-InterAct study.^1^

| **Food or beverage, portion size** | **Model** | **Relative difference in HRs (HR_1_/HR_0_)**^2^ **as a measure of multiplicative interaction** | | | | | | | | |
| --- | --- | --- | --- | --- | --- | --- | --- | --- | --- | --- |
|  |  | **uwPRS for type 2 diabetes** | | | **uwPRS for insulin resistance** | | | **uwPRS for body mass index**^3^ | | |
|  |  | **HR_1_/HR_0_ (95% CI)** | ***P*** | **I^2^, %** | **HR_1_/HR_0_ (95% CI)** | ***P*** | **I^2^, %** | **HR_1_/HR_0_ (95% CI)** | ***P*** | **I^2^, %** |
| Fruit, 100 g/d | *1* | 0.99 (0.96, 1.02) | 0.45 | 33.3 | 1.02 (1.00, 1.04) | 0.054 | 0.0 | 1.00 (0.98, 1.02) | 0.94 | 0.0 |
|  | *2* | 0.98 (0.96, 1.01) | 0.24 | 26.9 | 1.02 (1.00, 1.04) | 0.078 | 0.0 | 1.00 (0.98, 1.02) | 0.92 | 0.0 |
|  | *3* | 0.99 (0.97, 1.02) | 0.66 | 0.0 | 1.02 (0.99, 1.06) | 0.25 | 36.7 | ‡ |  |  |
| Green leafy vegetable,  90 g/d | *1* | 0.94 (0.78, 1.14) | 0.56 | 43.9 | 1.06 (0.94, 1.19) | 0.37 | 12.3 | 1.02 (0.93, 1.12) | 0.64 | 0.0 |
|  | *2* | 0.91 (0.78, 1.06) | 0.23 | 17.4 | 1.08 (0.94, 1.25) | 0.27 | 16.7 | 1.03 (0.93, 1.13) | 0.59 | 0.0 |
|  | *3* | 0.92 (0.83, 1.03) | 0.14 | 0.0 | 1.16 (0.91, 1.48) | 0.22 | 51.7 |  |  |  |
| Root vegetable, 80 g/d | *1* | 0.96 (0.83, 1.12) | 0.64 | 30.8 | 1.02 (0.90, 1.15) | 0.77 | 22.1 | 1.01 (0.93, 1.11) | 0.76 | 0.0 |
|  | *2* | 0.97 (0.86, 1.09) | 0.60 | 3.7 | 1.03 (0.94, 1.13) | 0.53 | 0.0 | 1.00 (0.91, 1.10) | 0.94 | 0.0 |
|  | *3* | 0.85 (0.69, 1.04) | 0.12 | 37.0 | 1.02 (0.86, 1.21) | 0.82 | 32.1 |  |  |  |
| Wholegrains, 40 g/d | *1* | 0.99 (0.96, 1.02) | 0.55 | 39.8 | 1.01 (0.98, 1.03) | 0.62 | 19.0 | 1.02 (0.99, 1.04) | 0.27 | 20.2 |
|  | *2* | 0.99 (0.95, 1.02) | 0.45 | 37.1 | 1.00 (0.97, 1.03) | 0.99 | 10.2 | 1.01 (0.98, 1.04) | 0.58 | 21.5 |
|  | *3* | 0.98 (0.94, 1.03) | 0.44 | 45.1 | 0.99 (0.97, 1.02) | 0.65 | 0.0 |  |  |  |
| Rice, 100 g/d | *1* | 0.98 (0.82, 1.17) | 0.82 | 14.5 | 1.10 (0.94, 1.28) | 0.24 | 0.0 | 0.98 (0.80, 1.20) | 0.83 | 36.4 |
|  | *2* | 0.96 (0.78, 1.17) | 0.67 | 19.0 | 1.17 (0.99, 1.38) | 0.07 | 0.0 | 0.98 (0.82, 1.17) | 0.81 | 14.2 |
|  | *3* | 1.04 (0.86, 1.25) | 0.69 | 0.0 | 1.27 (1.06, 1.54) | 0.011 | 0.0 |  |  |  |
| Legumes, 35 g/d^4^ | *1* | 0.99 (0.89, 1.10) | 0.81 | 42.9 | 1.01 (0.95, 1.08) | 0.71 | 0.0 | 0.94 (0.88, 1.01) | 0.072 | 4.3 |
|  | *2* | 0.92 (0.80, 1.05) | 0.22 | 59.4 | 1.02 (0.95, 1.09) | 0.64 | 0.0 | 0.95 (0.89, 1.01) | 0.089 | 0.0 |
|  | *3* | 0.91 (0.78, 1.05) | 0.19 | 54.5 | 0.97 (0.84, 1.11) | 0.65 | 47.7 |  |  |  |
| Nuts and seeds, 30 g/d | *1* | 0.92 (0.78, 1.08) | 0.29 | 0.0 | 1.05 (0.86, 1.28) | 0.62 | 10.9 | 0.94 (0.80, 1.12) | 0.50 | 0.0 |
|  | *2* | 0.83 (0.67, 1.03) | 0.09 | 14.7 | 1.08 (0.82, 1.43) | 0.57 | 36.0 | 0.90 (0.72, 1.12) | 0.35 | 20.1 |
|  | *3* | 0.75 (0.54, 1.05) | 0.09 | 45.6 | 0.95 (0.72, 1.25) | 0.72 | 18.1 |  |  |  |
| Fermented dairy,  125 ml/d | *1* | 1.01 (0.97, 1.06) | 0.59 | 0.0 | 1.01 (0.95, 1.06) | 0.83 | 17.5 | 0.99 (0.95, 1.04) | 0.74 | 0.0 |
|  | *2* | 1.02 (0.98, 1.08) | 0.33 | 0.0 | 1.00 (0.94, 1.07) | 0.98 | 29.7 | 0.99 (0.95, 1.04) | 0.71 | 0.0 |
|  | *3* | 1.03 (0.94, 1.13) | 0.53 | 50.3 | 0.97 (0.91, 1.04) | 0.42 | 23.0 |  |  |  |
| Red meat, 144 g/d | *1* | 0.96 (0.83, 1.12) | 0.63 | 6.2 | 1.15 (1.01, 1.31) | 0.032 | 0.0 | 0.95 (0.84, 1.08) | 0.47 | 0.0 |
|  | *2* | 1.02 (0.85, 1.23) | 0.80 | 20.6 | 1.19 (1.00, 1.41) | 0.047 | 18.7 | 0.96 (0.83, 1.11) | 0.57 | 0.0 |
|  | *3* | 1.06 (0.89, 1.25) | 0.51 | 0.0 | 1.22 (1.04, 1.42) | 0.014 | 0.0 |  |  |  |
| Processed red meat,  75 g/d | *1* | 0.97 (0.86, 1.10) | 0.66 | 41.0 | 1.03 (0.94, 1.14) | 0.51 | 29.6 | 1.01 (0.93, 1.08) | 0.89 | 0.0 |
|  | *2* | 0.95 (0.82, 1.09) | 0.44 | 46.6 | 1.04 (0.91, 1.18) | 0.58 | 45.5 | 1.01 (0.93, 1.10) | 0.77 | 0.0 |
|  | *3* | 0.87 (0.73, 1.05) | 0.14 | 58.8 | 1.04 (0.87, 1.24) | 0.68 | 63.3 |  |  |  |
| Fish, 100 g/d | *1* | 1.16 (0.79, 1.71) | 0.44 | 36.5 | 1.17 (0.92, 1.49) | 0.20 | 0.0 | 0.93 (0.68, 1.27) | 0.64 | 22.6 |
|  | *2* | 1.29 (0.81, 2.04) | 0.28 | 46.6 | 1.14 (0.88, 1.47) | 0.33 | 0.0 | 1.02 (0.68, 1.53) | 0.92 | 38.1 |
|  | *3* | 1.32 (0.77, 2.26) | 0.32 | 51.6 | 1.21 (0.85, 1.72) | 0.28 | 18.1 |  |  |  |
| Egg and egg products,  50 g/d | *1* | 0.96 (0.87, 1.06) | 0.38 | 0.0 | 1.12 (0.96, 1.31) | 0.14 | 49.7 | 1.02 (0.93, 1.12) | 0.65 | 0.0 |
|  | *2* | 0.99 (0.85, 1.16) | 0.92 | 33.3 | 1.08 (0.93, 1.27) | 0.30 | 37.7 | 1.05 (0.95, 1.16) | 0.33 | 0.0 |
|  | *3* | 0.98 (0.81, 1.18) | 0.81 | 42.0 | 1.07 (0.87, 1.31) | 0.52 | 54.4 |  |  |  |
| Sugar-sweetened beverages, 366 ml/d | *1* | 1.21 (1.04, 1.41) | 0.014 | 41.8 | 0.97 (0.86, 1.08) | 0.56 | 25.1 | 0.96 (0.88, 1.05) | 0.39 | 0.0 |
|  | *2* | 1.27 (1.05, 1.52) | 0.012 | 54.2 | 0.97 (0.87, 1.07) | 0.52 | 4.6 | 0.97 (0.87, 1.07) | 0.50 | 0.0 |
|  | *3* | 1.28 (1.03, 1.60) | 0.028 | 60.2 | 1.00 (0.84, 1.19) | 0.99 | 43.2 |  |  |  |
| Coffee, 260 ml/d | *1* | 1.00 (0.96, 1.05) | 0.86 | 48.7 | 1.02 (0.99, 1.05) | 0.13 | 0.0 | 1.01 (0.98, 1.03) | 0.53 | 0.0 |
|  | *2* | 0.99 (0.94, 1.04) | 0.60 | 45.0 | 1.02 (0.99, 1.05) | 0.19 | 0.0 | 1.01 (0.99, 1.04) | 0.37 | 0.0 |
|  | *3* | 0.99 (0.94, 1.05) | 0.82 | 44.9 | 1.02 (0.99, 1.05) | 0.29 | 0.0 |  |  |  |
| Tea, 260 ml/d | *1* | 0.99 (0.96, 1.03) | 0.66 | 0.0 | 0.97 (0.94, 1.01) | 0.12 | 0.0 | 1.00 (0.97, 1.03) | 0.92 | 0.0 |
|  | *2* | 0.98 (0.94, 1.02) | 0.31 | 0.0 | 0.98 (0.94, 1.03) | 0.43 | 8.9 | 0.98 (0.95, 1.02) | 0.32 | 0.0 |
|  | *3* | 0.99 (0.96, 1.03) | 0.66 | 0.0 | 0.97 (0.94, 1.01) | 0.12 | 0.0 |  |  |  |

^1^ N cases=9,542 incident T2D cases, N sample=21,437.

^2^ A beta coefficient (β) for the interaction between each unweighted PRS and each of the foods and beverages on incident T2D was estimated per one unit difference for each PRS and food/beverage. One unit of PRS was 1 standard deviation in the EPIC InterAct subgroup, and one unit of foods/beverages was a standard portion size for each, as written on the first column. Thus, exp(β), for example, represented a ratio of two hazard ratios (HR_1_/HR_0_) consisting of HR_1_ per 1 portion of food/beverage when PRS=+1 SD and HR_0_ per 1 portion of food/beverage when PRS=0. Estimates were adjusted for age (=underlying time scale), sex, centre, total energy intake, first 5 principal components for population stratification (Model 1); model 1 + physical activity, education, smoking, sex-specific alcohol categories, and other food/beverage consumption listed (Model 2); and model 2+ baseline BMI (Model 3). As exception, legume consumption was not evaluated and not adjusted for in Denmark. §
^3^ The analyses using the BMI PRS did not adjust for baseline BMI.

^4^ N=7,749 incident T2D cases/17,880 total participants, as legume consumption was not assessed in Denmark.

# Supplemental Table 4. Additive interaction between three weighted polygenic risk scores (wPRSs) and food or beverage intakes on incident type 2 diabetes: EPIC-InterAct study.^1^

| **Food or beverage, portion size** | **Relative excess risk for interaction (RERI) as a measure of additive interaction** | | | | | | | | |
| --- | --- | --- | --- | --- | --- | --- | --- | --- | --- |
|  | **wPRS for type 2 diabetes** | | | **wPRS for insulin resistance** | | | **wPRS for body mass index**^2^ | | |
|  | **RERI (95% CI)** | ***P*** | **I^2^, %** | **RERI (95% CI)** | ***P*** | **I^2^, %** | **RERI (95% CI)** | ***P*** | **I^2^, %** |
| Fruit, 100 g/d | -0.01 (-0.06, 0.05) | 0.78 | 23.0 | 0.01 (-0.01, 0.04) | 0.41 | 2.0 | 0.00 (-0.03, 0.02) | 0.76 | 0.0 |
| Green leafy vegetables, 90 g/d | -0.35 (-0.80, 0.11) | 0.14 | 45.3 | 0.02 (-0.09, 0.12) | 0.73 | 0.0 | 0.02 (-0.10, 0.14) | 0.73 | 0.0 |
| Root vegetables, 80 g/d | -0.44 (-0.82, -0.07) | 0.02 | 67.2 | -0.03 (-0.14, 0.08) | 0.57 | 11.5 | -0.04 (-0.13, 0.05) | 0.42 | 0.0 |
| Wholegrains, 40 g/d | 0.00 (-0.07, 0.06) | 0.92 | 20.0 | -0.01 (-0.05, 0.02) | 0.41 | 35.5 | 0.01 (-0.03, 0.04) | 0.65 | 19.4 |
| Rice, 100 g/d | -0.53 (-0.88, -0.17) | 0.004 | 60.7 | -0.01 (-0.12, 0.10) | 0.89 | 0.0 | -0.10 (-0.27, 0.07) | 0.23 | 0.0 |
| Fermented dairy, 125 ml/d | -0.04 (-0.13, 0.06) | 0.46 | 0.0 | -0.03 (-0.09, 0.02) | 0.22 | 0.0 | -0.01 (-0.06, 0.04) | 0.64 | 0.0 |
| Nuts and seeds, 30 g/d | -0.47 (-0.89, -0.06) | 0.03 | 18.0 | -0.11 (-0.29, 0.07) | 0.25 | 0.0 | -0.20 (-0.37, -0.03) | 0.023 | 0.0 |
| Legumes, 35 g/d^3^ | -0.11 (-0.29, 0.07) | 0.22 | 12.5 | -0.01 (-0.13, 0.10) | 0.81 | 20.6 | -0.08 (-0.14, -0.01) | 0.020 | 0.0 |
| Red meat, 144 g/d | -0.11 (-0.68, 0.47) | 0.71 | 50.8 | 0.19 (-0.04, 0.41) | 0.10 | 0.0 | 0.02 (-0.21, 0.25) | 0.84 | 0.0 |
| Processed meat, 75 g/d | 0.08 (-0.19, 0.36) | 0.55 | 24.1 | 0.02 (-0.13, 0.16) | 0.83 | 26.8 | 0.06 (-0.07, 0.20) | 0.37 | 0.0 |
| Fish, 100 g/d | 0.26 (-0.55, 1.07) | 0.52 | 9.4 | -0.01 (-0.33, 0.32) | 0.97 | 0.0 | -0.30 (-0.57, -0.02) | 0.033 | 0.0 |
| Egg and egg products, 50 g/d | 0.02 (-0.23, 0.27) | 0.86 | 0.0 | 0.02 (-0.22, 0.26) | 0.86 | 49.0 | 0.04 (-0.11, 0.19) | 0.59 | 0.0 |
| Sugar-sweetened beverages, 336 ml/d | 0.32 (-0.15, 0.80) | 0.19 | 48.3 | -0.15 (-0.29, -0.01) | 0.04 | 22.7 | -0.02 (-0.14, 0.11) | 0.81 | 0.0 |
| Coffee, 260 ml/d | -0.12 (-0.21, -0.03) | 0.009 | 41.6 | 0.02 (-0.02, 0.05) | 0.32 | 0.0 | 0.01 (-0.03, 0.05) | 0.56 | 27.0 |
| Tea, 260 ml/d | -0.13 (-0.27, 0.02) | 0.09 | 55.3 | -0.04 (-0.07, 0.00) | 0.05 | 0.0 | -0.03 (-0.07, 0.00) | 0.074 | 0.0 |

^1^ N cases=9,542 incident T2D cases, N sample=21,437. RERI for the interaction between each weighted PRS and each of the foods and beverages on incident T2D was estimated per one unit difference for each PRS and each food/beverage, using parameter estimates obtained from Prentice-weighted Cox proportional hazard regression. One unit of PRS was 1 standard deviation in the EPIC InterAct subcohort, and one unit of foods/beverages was a standard portion size for each, as written on the first column. Estimates were adjusted for age (=underlying time scale), sex, centre, total energy intake, first 5 principal components for population stratification, physical activity, education, smoking, sex-specific alcohol categories, and other food/beverage consumption listed, and baseline BMI. As exception, legume consumption was not evaluated and not adjusted for in Denmark.
^2^ The analyses using the BMI PRS did not adjust for baseline BMI.

^3^ N=7,749 incident T2D cases/17,880 total participants.


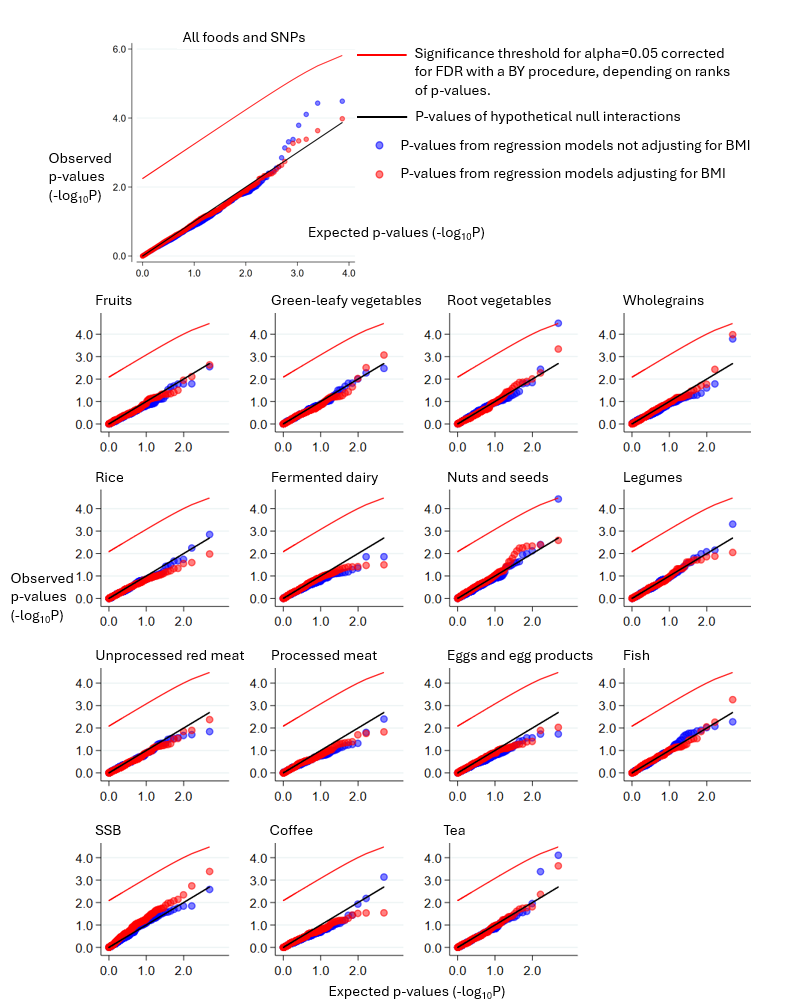


Supplemental Figure 3. Q-Q plots of results from tests for multiplicative interactions between 15 food items and 247 single nucleotide polymorphisms contributing to the polygenic risk scores: EPIC-InterAct case-cohort analysis. Country- and gene-chip specific prentice-weighted multivariable-adjusted Cox regression model was fitted (N=21,437; 9,542 cases and 11,895 non-cases), followed by random-effects meta-analysis. Blue dots represent -log_10_(p-values) when not adjusted for BMI; and red dots, when adjusted for BMI. Each red line represents significant thresholds across the ranks of p-values from the multiple tests that underwent a Benjamini–Yekutieli (BY) correction for the false-discovery rates (FDR). The top panel represents the Q-Q plot of all the interaction results of 15 food items and 247 single nucleotide polymorphisms (SNPs). Smaller plots represent those of individual food items and 247 SNPs. SSB stands for sugar-sweetened beverages.

| Supplemental Table 5. Single nucleotide polymorphisms (SNPs) with a potential interaction (crude p<0.05) with one or more of 15 food items for incidence of type 2 diabetes: EPIC-InterAct case-cohort analysis^1^. | | | | | | | | | | |  |
| --- | --- | --- | --- | --- | --- | --- | --- | --- | --- | --- | --- |
| SNPs | Food group | BMI adj^2^ | HR_1_/HR_0_ (95% CI)^3^ | *I^2^* | -log_10_*P* ^4^ | Chromosome: position | Allele | Freq^5^ | Gene symbol^6^ | Function^6^ | |
| rs4929949 | Root vegetables |  | 1.36 (1.18, 1.57) | 0.0 | 4.49 | 11:8604593 | T/C | 0.49 | STK33 | intronic, non-coding intronic | |
|  | Root vegetables | x | 1.33 (1.09, 1.62) | 13.7 | 2.27 |  |  |  |  |  | |
| rs2197076 | Nuts and seeds |  | 2.05 (1.46, 2.89) | 4.5 | 4.43 | 2:88422758 | G/A | 0.19 | FABP1 | 3utr, intronic, non-coding | |
|  | Nuts and seeds | x | 1.56 (1.09, 2.25) | 0.0 | 1.80 |  |  |  |  |  | |
|  | SSB^7^ | x | 0.82 (0.68, 0.99) | 0.0 | 1.38 |  |  |  |  |  | |
| rs6066149 | Tea |  | 1.13 (1.06, 1.20) | 0.0 | 4.11 | 20:45602638 | G/A | 0.25 | EYA2 | intronic | |
|  | Legumes |  | 1.15 (1.04, 1.28) | 0.0 | 2.08 |  |  |  |  |  | |
|  | Tea | x | 1.13 (1.02, 1.26) | 30.5 | 1.75 |  |  |  |  |  | |
|  | Rice | x | 0.63 (0.41, 0.95) | 41.5 | 1.55 |  |  |  |  |  | |
| rs6878122 | Wholegrains | x | 0.92 (0.88, 0.96) | 0.0 | 3.98 | 5:76427311 | G/A | 0.69 | ZBED3-AS1 | 3downstream, non-coding intronic | |
|  | Wholegrains |  | 0.93 (0.90, 0.97) | 0.0 | 3.79 |  |  |  |  |  | |
|  | Green vegetables | x | 0.81 (0.70, 0.93) | 0.0 | 2.51 |  |  |  |  |  | |
|  | Fish |  | 0.68 (0.46, 1.00) | 0.0 | 1.32 |  |  |  |  |  | |
| rs11063069 | Tea | x | 1.13 (1.06, 1.21) | 0.0 | 3.63 | 12:4374373 | A/G | 0.21 | CCND2-AS1 | non-coding intronic | |
|  | Tea |  | 1.11 (1.05, 1.18) | 0.0 | 3.38 |  |  |  |  |  | |
| rs1441264 | SSB | x | 1.40 (1.16, 1.69) | 14.5 | 3.38 | 13:79580919 | G/A | 0.61 |  |  | |
|  | SSB |  | 1.25 (1.08, 1.45) | 0.0 | 2.58 |  |  |  |  |  | |
| rs6091540 | Root vegetables | x | 1.38 (1.15, 1.65) | 0.0 | 3.34 | 20:51087862 | C/T | 0.27 | RP4-723E3.1 | non-coding intronic | |
|  | Processed meat | x | 0.83 (0.72, 0.97) | 0.0 | 1.83 |  |  |  |  |  | |
|  | Root vegetables |  | 1.23 (1.04, 1.45) | 0.0 | 1.79 |  |  |  |  |  | |
|  | Processed meat |  | 0.88 (0.77, 1.00) | 0.0 | 1.32 |  |  |  |  |  | |
| rs12899811 | Legumes |  | 1.18 (1.08, 1.30) | 0.0 | 3.31 | 15:91544076 | A/G | 0.31 | VPS33B | intronic, non-coding, non-coding intronic | |
|  | Legumes | x | 1.20 (1.04, 1.38) | 9.4 | 1.88 |  |  |  |  |  | |
|  | Fish |  | 1.51 (1.02, 2.24) | 0.0 | 1.41 |  |  |  |  |  | |
| rs4740619 | Fish | x | 1.97 (1.34, 2.90) | 0.0 | 3.27 | 9:15634326 | T/C | 0.45 | CCDC171 | intronic | |
|  | Fish |  | 1.59 (1.12, 2.26) | 0.0 | 2.02 |  |  |  |  |  | |
|  | Nuts and seeds | x | 0.67 (0.47, 0.98) | 17.1 | 1.43 |  |  |  |  |  | |
| rs2820292 | Coffee |  | 1.07 (1.03, 1.11) | 0.0 | 3.13 | 1:201784287 | A/C | 0.55 | NAV1 | 3downstream, intronic, non-coding intronic | |
|  | Legumes |  | 1.29 (1.03, 1.61) | 53.5 | 1.61 |  |  |  |  |  | |
|  | Coffee | x | 1.09 (1.01, 1.17) | 43.7 | 1.54 |  |  |  |  |  | |
| rs10278336 | Green vegetables | x | 0.78 (0.68, 0.90) | 0.0 | 3.07 | 7:44245363 | A/G | 0.41 | YKT6 | 5upstream, intronic, non-coding intronic | |
|  | Green vegetables |  | 0.83 (0.72, 0.95) | 0.0 | 2.27 |  |  |  |  |  | |
|  | Rice |  | 1.30 (1.04, 1.62) | 0.0 | 1.67 |  |  |  |  |  | |
|  | Nuts and seeds | x | 1.31 (1.00, 1.71) | 0.0 | 1.32 |  |  |  |  |  | |
| rs7202877 | Rice |  | 0.57 (0.40, 0.80) | 0.0 | 2.85 | 16:75247245 | T/G | 0.10 |  |  | |
|  | Unprocessed red meat |  | 0.64 (0.45, 0.93) | 13.4 | 1.71 |  |  |  |  |  | |
|  | Rice | x | 0.52 (0.29, 0.92) | 36.6 | 1.60 |  |  |  |  |  | |
|  | Egg/egg products |  | 0.76 (0.59, 0.97) | 8.3 | 1.56 |  |  |  |  |  | |
| rs7899106 | SSB | x | 2.18 (1.34, 3.56) | 35.3 | 2.74 | 10:87410904 | A/G | 0.05 | GRID1 | 5upstream, intronic | |
|  | Nuts and seeds | x | 2.29 (1.25, 4.22) | 4.2 | 2.11 |  |  |  |  |  | |
|  | Wholegrains | x | 1.12 (1.01, 1.24) | 19.5 | 1.58 |  |  |  |  |  | |
|  | SSB |  | 1.93 (1.08, 3.44) | 61.4 | 1.57 |  |  |  |  |  | |
| rs1045241 | Fruits | x | 0.94 (0.91, 0.98) | 0.0 | 2.63 | 5:118729286 | C/T | 0.28 | TNFAIP8 | 3downstream, 3utr | |
| rs6567160 | Nuts and seeds | x | 1.60 (1.18, 2.17) | 0.0 | 2.59 | 18:57829135 | T/C | 0.24 | RNU4-17P | 3downstream, 5upstream | |
| rs10132280 | Fruits |  | 0.95 (0.93, 0.98) | 0.0 | 2.55 | 14:25928179 | C/A | 0.31 |  |  | |
|  | Root vegetables | x | 1.26 (1.04, 1.53) | 0.0 | 1.68 |  |  |  |  |  | |
| rs498313 | Green vegetables |  | 0.81 (0.70, 0.93) | 0.0 | 2.47 | 9:78034169 | A/G | 0.31 |  |  | |
| rs4836133_C_A | Root vegetables |  | 0.75 (0.61, 0.91) | 21.7 | 2.44 | 5:124332103 | C/A | 0.48 |  |  | |
|  | Root vegetables | x | 0.78 (0.63, 0.95) | 13.6 | 1.86 |  |  |  |  |  | |
|  | SSB |  | 0.83 (0.72, 0.96) | 6.4 | 1.85 |  |  |  |  |  | |
|  | SSB | x | 0.67 (0.48, 0.94) | 74.7 | 1.68 |  |  |  |  |  | |
| rs2365389 | Wholegrains | x | 0.93 (0.89, 0.98) | 16.7 | 2.43 | 3:61236462 | C/T | 0.40 | FHIT | intronic, non-coding intronic | |
|  | Wholegrains |  | 0.96 (0.93, 1.00) | 0.0 | 1.60 |  |  |  |  |  | |
| rs11130329 | Nuts and seeds |  | 1.68 (1.18, 2.39) | 0.0 | 2.40 | 3:52896855 | C/A | 0.84 | TMEM110 | intronic | |
|  | Legumes |  | 1.36 (1.02, 1.81) | 49.1 | 1.41 |  |  |  |  |  | |
|  | Legumes | x | 1.45 (1.00, 2.09) | 54.7 | 1.33 |  |  |  |  |  | |
| rs16907751 | Processed meat |  | 0.77 (0.64, 0.92) | 0.0 | 2.40 | 8:81375457 | C/T | 0.11 |  |  | |
|  | Green vegetables |  | 0.76 (0.62, 0.94) | 0.0 | 2.00 |  |  |  |  |  | |
|  | SSB | x | 1.42 (1.02, 1.98) | 29.3 | 1.40 |  |  |  |  |  | |
| rs571312 | Nuts and seeds | x | 1.55 (1.15, 2.10) | 0.0 | 2.39 | 18:57839769 | C/A | 0.24 |  |  | |
| rs2080454 | Unprocessed red meat | x | 0.71 (0.56, 0.90) | 0.0 | 2.38 | 16:49062590 | C/A | 0.63 |  |  | |
|  | Unprocessed red meat |  | 0.77 (0.63, 0.95) | 0.0 | 1.84 |  |  |  |  |  | |
| rs459193 | Tea | x | 0.91 (0.85, 0.97) | 0.0 | 2.36 | 5:55806751 | A/G | 0.74 | AC022431.2 | 3downstream | |
|  | Processed meat | x | 0.86 (0.74, 0.99) | 0.0 | 1.39 |  |  |  |  |  | |
| rs10195252 | SSB | x | 1.24 (1.07, 1.44) | 0.0 | 2.34 | 2:165513091 | T/C | 0.41 | COBLL1 | non-coding intronic | |
|  | SSB |  | 1.15 (1.01, 1.32) | 0.0 | 1.48 |  |  |  |  |  | |
| rs13078807 | Nuts and seeds | x | 1.61 (1.16, 2.24) | 0.0 | 2.34 | 3:85884150 | A/G | 0.20 | CADM2 | intronic | |
|  | Nuts and seeds |  | 1.46 (1.09, 1.95) | 0.0 | 1.95 |  |  |  |  |  | |
|  | Processed meat | x | 1.47 (1.01, 2.13) | 78.2 | 1.36 |  |  |  |  |  | |
| rs13078960 | Nuts and seeds | x | 1.64 (1.16, 2.31) | 2.3 | 2.33 | 3:85807590 | T/G | 0.20 | CADM2 | intronic | |
|  | Nuts and seeds |  | 1.46 (1.02, 2.08) | 14.9 | 1.43 |  |  |  |  |  | |
|  | Processed meat | x | 1.46 (1.01, 2.12) | 77.9 | 1.34 |  |  |  |  |  | |
| rs132985 | Fish |  | 0.60 (0.42, 0.86) | 0.0 | 2.28 | 22:38563471 | C/T | 0.47 | PLA2G6 | 3downstream, intronic, non-coding intronic | |
|  | Fish | x | 0.57 (0.34, 0.95) | 22.9 | 1.49 |  |  |  |  |  | |
|  | Fermented Dairy | x | 1.08 (1.00, 1.16) | 0.0 | 1.42 |  |  |  |  |  | |
| rs6795735 | Fish | x | 1.78 (1.18, 2.67) | 0.0 | 2.26 | 3:64705365 | C/T | 0.42 | ADAMTS9-AS2 | non-coding intronic | |
|  | Fish |  | 1.66 (1.14, 2.43) | 0.0 | 2.08 |  |  |  |  |  | |
| rs13107325 | Nuts and seeds | x | 2.33 (1.28, 4.24) | 0.0 | 2.26 | 4:103188709 | C/T | 0.06 | SLC39A8 | **Coding (391: A>S\|A>T)** | |
|  | Unprocessed red meat |  | 0.56 (0.34, 0.94) | 24.1 | 1.53 |  |  |  |  |  | |
|  | Nuts and seeds |  | 2.33 (1.05, 5.18) | 28.2 | 1.42 |  |  |  |  |  | |
| rs2033732 | Rice |  | 0.69 (0.53, 0.90) | 0.0 | 2.25 | 8:85079709 | T/C | 0.76 |  |  | |
|  | Green vegetables | x | 0.82 (0.68, 0.99) | 0.0 | 1.37 |  |  |  |  |  | |
|  | Rice | x | 0.74 (0.55, 1.00) | 0.0 | 1.33 |  |  |  |  |  | |
| rs1800574 | Nuts and seeds | x | 5.54 (1.65, 18.64) | 33.0 | 2.24 | 12:121416864 | C/T | 0.03 | HNF1A-AS1 | **Coding (146, 98: A>V)**, intronic, non-coding intronic | |
|  | Nuts and seeds |  | 3.93 (1.44, 10.74) | 13.5 | 2.12 |  |  |  |  |  | |
| rs205262 | Coffee |  | 1.06 (1.02, 1.11) | 0.0 | 2.18 | 6:34563164 | A/G | 0.28 | C6orf106 | intronic | |
|  | Coffee | x | 1.07 (1.00, 1.14) | 25.3 | 1.44 |  |  |  |  |  | |
|  | Unprocessed red meat |  | 0.80 (0.64, 1.00) | 0.0 | 1.32 |  |  |  |  |  | |
| rs3810291 | Legumes |  | 1.13 (1.03, 1.24) | 0.0 | 2.15 | 19:47569003 | G/A | 0.68 | ZC3H4 | 3utr, non-coding | |
| rs1928295 | SSB | x | 0.79 (0.66, 0.94) | 17.0 | 2.14 | 9:120378483 | T/C | 0.45 |  |  | |
|  | SSB |  | 0.87 (0.76, 0.99) | 0.0 | 1.49 |  |  |  |  |  | |
| rs17094222 | Fruits | x | 1.05 (1.01, 1.10) | 0.0 | 2.11 | 10:102395440 | T/C | 0.20 |  |  | |
|  | Fish | x | 1.85 (1.13, 3.04) | 0.0 | 1.84 |  |  |  |  |  | |
|  | SSB | x | 1.40 (1.03, 1.90) | 48.9 | 1.49 |  |  |  |  |  | |
|  | Fish |  | 1.72 (1.04, 2.83) | 8.6 | 1.47 |  |  |  |  |  | |
| rs13389219 | SSB | x | 1.28 (1.07, 1.55) | 21.9 | 2.08 | 2:165528876 | C/T | 0.41 | COBLL1 | non-coding intronic | |
|  | SSB |  | 1.16 (1.02, 1.33) | 0.0 | 1.53 |  |  |  |  |  | |
|  | Tea | x | 1.14 (1.00, 1.29) | 60.1 | 1.33 |  |  |  |  |  | |
| rs3849570 | Fish | x | 1.71 (1.14, 2.54) | 0.0 | 2.05 | 3:81792112 | C/A | 0.34 | GBE1 | intronic | |
|  | Fish |  | 1.59 (1.10, 2.29) | 0.0 | 1.87 |  |  |  |  |  | |
|  | Unprocessed red meat |  | 1.25 (1.02, 1.54) | 0.0 | 1.49 |  |  |  |  |  | |
| rs3736485 | Legumes | x | 0.88 (0.80, 0.97) | 0.0 | 2.05 | 15:51748610 | A/G | 0.54 | DMXL2 | 3downstream, 5upstream, intronic, non-coding intronic | |
|  | Nuts and seeds |  | 0.70 (0.53, 0.92) | 6.5 | 1.99 |  |  |  |  |  | |
|  | Legumes |  | 0.90 (0.82, 0.98) | 0.0 | 1.80 |  |  |  |  |  | |
| rs17024393 | Egg/egg products | x | 1.80 (1.16, 2.81) | 0.0 | 2.03 | 1:110154688 | T/C | 0.03 | GNAT2 | intronic | |
|  | Root vegetables | x | 2.37 (1.20, 4.68) | 27.5 | 1.89 |  |  |  |  |  | |
|  | Legumes | x | 1.45 (1.05, 2.00) | 0.0 | 1.63 |  |  |  |  |  | |
| rs754814 | Green vegetables | x | 0.81 (0.70, 0.95) | 0.0 | 2.03 | 17:4657034 | C/T | 0.72 |  |  | |
|  | Green vegetables |  | 0.83 (0.72, 0.97) | 0.0 | 1.82 |  |  |  |  |  | |
|  | Legumes |  | 1.13 (1.02, 1.25) | 0.0 | 1.81 |  |  |  |  |  | |
|  | Fish | x | 1.66 (1.05, 2.61) | 0.0 | 1.53 |  |  |  |  |  | |
|  | Nuts and seeds |  | 1.36 (1.01, 1.81) | 1.9 | 1.39 |  |  |  |  |  | |
| rs3736265 | Root vegetables | x | 0.60 (0.40, 0.88) | 0.0 | 2.01 | 4:23814707 | G/A | 0.06 | PPARGC1A | 3downstream, 3utr, **coding (612: T>M\| T>K)**, non-coding | |
|  | Root vegetables |  | 0.69 (0.49, 0.98) | 0.0 | 1.45 |  |  |  |  |  | |
|  | SSB | x | 1.49 (1.01, 2.21) | 33.0 | 1.33 |  |  |  |  |  | |
| rs7599312 | SSB | x | 1.32 (1.07, 1.63) | 24.6 | 1.99 | 2:213413231 | G/A | 0.27 |  |  | |
|  | SSB |  | 1.20 (1.04, 1.38) | 0.0 | 1.84 |  |  |  |  |  | |
|  | Tea |  | 1.06 (1.01, 1.12) | 0.0 | 1.55 |  |  |  |  |  | |
| rs4804311 | Legumes |  | 0.85 (0.76, 0.96) | 0.0 | 1.99 | 19:8615589 | A/G | 0.10 | MYO1F | 3downstream, 5upstream, intronic, non-coding intronic | |
|  | Legumes | x | 0.86 (0.76, 0.98) | 0.0 | 1.62 |  |  |  |  |  | |
| rs11577194 | Rice | x | 0.72 (0.56, 0.93) | 0.0 | 1.98 | 1:110500175 | T/C | 0.53 |  |  | |
| rs7177055 | SSB | x | 0.80 (0.67, 0.95) | 6.5 | 1.97 | 15:77832762 | G/A | 0.71 |  |  | |
|  | Tea |  | 1.08 (1.02, 1.14) | 0.0 | 1.96 |  |  |  |  |  | |
|  | Unprocessed red meat | x | 0.72 (0.56, 0.93) | 0.0 | 1.90 |  |  |  |  |  | |
|  | Tea | x | 1.10 (1.02, 1.18) | 10.1 | 1.81 |  |  |  |  |  | |
|  | Unprocessed red meat |  | 0.75 (0.59, 0.96) | 11.1 | 1.67 |  |  |  |  |  | |
| rs12525532 | Nuts and seeds | x | 1.44 (1.09, 1.90) | 0.0 | 1.96 | 6:35004819 | C/T | 0.38 | ANKS1A | intronic | |
| rs1528435 | Fruits | x | 0.96 (0.93, 0.99) | 0.0 | 1.95 | 2:181550962 | C/T | 0.62 | AC009478.1 | non-coding intronic | |
| rs560887 | Coffee |  | 0.95 (0.91, 0.99) | 0.0 | 1.93 | 2:169763148 | T/C | 0.71 | SPC25 | intronic, non-coding intronic | |
|  | Coffee | x | 0.94 (0.89, 0.99) | 9.5 | 1.52 |  |  |  |  |  | |
| rs17036328 | Fish |  | 1.97 (1.16, 3.36) | 0.0 | 1.90 | 3:12390484 | T/C | 0.12 | PPARG | intronic, non-coding intronic | |
|  | Fruits |  | 1.07 (1.01, 1.13) | 17.3 | 1.78 |  |  |  |  |  | |
|  | Fruits | x | 1.07 (1.00, 1.13) | 16.9 | 1.40 |  |  |  |  |  | |
| rs12686676 | Egg/egg products | x | 1.57 (1.10, 2.25) | 72.7 | 1.89 | 9:102632493 | A/G | 0.56 |  |  | |
|  | Egg/egg products |  | 1.23 (1.01, 1.49) | 32.4 | 1.44 |  |  |  |  |  | |
| rs2334499 | SSB | x | 1.39 (1.07, 1.81) | 53.2 | 1.88 | 11:1696849 | C/T | 0.43 |  |  | |
|  | SSB |  | 1.19 (1.03, 1.38) | 0.0 | 1.76 |  |  |  |  |  | |
| rs849135 | Fermented Dairy |  | 0.92 (0.86, 0.98) | 0.0 | 1.86 | 7:28196413 | G/A | 0.48 | JAZF1 | intronic | |
|  | Fermented Dairy | x | 0.92 (0.85, 0.99) | 0.0 | 1.50 |  |  |  |  |  | |
| rs2126259 | Legumes | x | 0.81 (0.68, 0.96) | 0.0 | 1.86 | 8:9185146 | T/C | 0.91 | RP11-115J16.1 | 3downstream, non-coding intronic | |
| rs683135 | Fermented Dairy |  | 1.09 (1.02, 1.17) | 0.0 | 1.86 | 1:39895460 | G/A | 0.31 | MACF1 | 3downstream, intronic | |
|  | Tea | x | 1.10 (1.00, 1.21) | 27.9 | 1.40 |  |  |  |  |  | |
| rs7756992 | Root vegetables | x | 0.72 (0.55, 0.94) | 29.1 | 1.85 | 6:20679709 | A/G | 0.28 | CDKAL1 | intronic | |
|  | Fish |  | 0.38 (0.17, 0.84) | 56.9 | 1.77 |  |  |  |  |  | |
|  | Root vegetables |  | 0.84 (0.71, 0.99) | 0.0 | 1.38 |  |  |  |  |  | |
| rs11231693 | Unprocessed red meat | x | 0.55 (0.34, 0.89) | 0.0 | 1.85 | 11:63862612 | G/A | 0.06 | MACROD1 | intronic, non-coding intronic | |
|  | Unprocessed red meat |  | 0.65 (0.42, 1.00) | 0.0 | 1.32 |  |  |  |  |  | |
| rs308971 | Root vegetables |  | 1.29 (1.05, 1.59) | 0.0 | 1.84 | 3:12116620 | G/A | 0.87 | SYN2 | non-coding intronic | |
| rs10830963 | Root vegetables |  | 0.82 (0.70, 0.96) | 0.0 | 1.82 | 11:92708710 | C/G | 0.29 | MTNR1B | intronic | |
|  | Green vegetables |  | 1.72 (1.06, 2.82) | 64.0 | 1.53 |  |  |  |  |  | |
| rs9914578 | Green vegetables |  | 0.81 (0.68, 0.96) | 1.1 | 1.81 | 17:2005136 | C/G | 0.20 | SMG6 | 3downstream, intronic, non-coding intronic | |
|  | Nuts and seeds |  | 0.68 (0.49, 0.95) | 0.0 | 1.64 |  |  |  |  |  | |
| rs1167827 | Processed meat |  | 1.19 (1.03, 1.37) | 21.4 | 1.81 | 7:75163169 | A/G | 0.57 | HIP1 | 3downstream, 3utr | |
|  | Nuts and seeds |  | 1.46 (1.05, 2.04) | 24.5 | 1.60 |  |  |  |  |  | |
|  | Nuts and seeds | x | 1.67 (1.03, 2.70) | 52.4 | 1.44 |  |  |  |  |  | |
| rs2270188 | Fruits |  | 0.97 (0.94, 0.99) | 0.0 | 1.79 | 7:116140524 | G/T | 0.48 | CAV2 | 3utr, 5upstream, intronic, non-coding intronic | |
| rs1801282 | Fish |  | 1.97 (1.13, 3.43) | 0.0 | 1.79 | 3:12393125 | C/G | 0.11 | PPARG | **Coding (12: P>A)**, intronic, non-coding, non-coding intronic | |
|  | Fruits |  | 1.07 (1.01, 1.13) | 19.8 | 1.65 |  |  |  |  |  | |
|  | Fruits | x | 1.07 (1.00, 1.14) | 22.7 | 1.33 |  |  |  |  |  | |
| rs7164727 | Wholegrains |  | 1.05 (1.01, 1.09) | 11.7 | 1.78 | 15:73093991 | C/T | 0.70 |  |  | |
| rs2836754 | Fruits |  | 1.04 (1.01, 1.07) | 0.0 | 1.78 | 21:40291740 | T/C | 0.64 | AF064858.6 | non-coding intronic | |
| rs1516725 | Tea | x | 0.91 (0.84, 0.98) | 0.0 | 1.77 | 3:185824004 | T/C | 0.87 | ETV5 | 5upstream, intronic, non-coding intronic | |
|  | SSB | x | 0.66 (0.46, 0.94) | 52.3 | 1.70 |  |  |  |  |  | |
| rs2699429 | Fish |  | 1.55 (1.08, 2.21) | 0.0 | 1.77 | 4:3480136 | T/C | 0.43 | DOK7 | 3downstream, intronic, non-coding intronic | |
| rs11165643 | Wholegrains | x | 0.94 (0.90, 0.99) | 22.3 | 1.77 | 1:96924097 | C/T | 0.58 |  |  | |
|  | Green vegetables | x | 0.81 (0.68, 0.97) | 2.7 | 1.65 |  |  |  |  |  | |
|  | Green vegetables |  | 0.86 (0.75, 0.99) | 0.0 | 1.52 |  |  |  |  |  | |
| rs16851483 | SSB | x | 1.54 (1.08, 2.19) | 26.7 | 1.76 | 3:141275436 | G/T | 0.06 | RASA2 | intronic | |
| rs174550 | Processed meat | x | 0.85 (0.74, 0.97) | 0.0 | 1.76 | 11:61571478 | T/C | 0.32 | FADS1 | 5utr, intronic, non-coding intronic | |
|  | Green vegetables |  | 1.65 (1.08, 2.52) | 56.1 | 1.68 |  |  |  |  |  | |
|  | Green vegetables | x | 1.79 (1.04, 3.09) | 64.7 | 1.43 |  |  |  |  |  | |
|  | Root vegetables | x | 0.83 (0.70, 0.99) | 0.0 | 1.42 |  |  |  |  |  | |
| rs10946398 | Root vegetables | x | 0.79 (0.65, 0.96) | 3.2 | 1.75 | 6:20661034 | A/C | 0.32 | CDKAL1 | intronic | |
|  | Fish |  | 0.43 (0.21, 0.87) | 51.1 | 1.71 |  |  |  |  |  | |
|  | Root vegetables |  | 0.85 (0.72, 1.00) | 0.0 | 1.33 |  |  |  |  |  | |
| rs11651052 | SSB | x | 0.82 (0.70, 0.97) | 1.8 | 1.75 | 17:36102381 | A/G | 0.51 | HNF1B | intronic | |
| rs163184 | Rice |  | 0.63 (0.43, 0.92) | 54.1 | 1.74 | 11:2847069 | T/G | 0.49 | KCNQ1 | intronic, non-coding intronic | |
|  | Fish |  | 1.56 (1.07, 2.28) | 0.0 | 1.68 |  |  |  |  |  | |
| rs3101336 | Egg/egg products |  | 0.84 (0.73, 0.97) | 0.0 | 1.73 | 1:72751185 | T/C | 0.62 |  |  | |
|  | Legumes | x | 0.88 (0.80, 0.98) | 0.0 | 1.72 |  |  |  |  |  | |
|  | Rice |  | 1.28 (1.02, 1.62) | 0.0 | 1.43 |  |  |  |  |  | |
| rs2815752 | Egg/egg products |  | 0.84 (0.73, 0.97) | 0.0 | 1.73 | 1:72812440 | G/A | 0.62 |  |  | |
|  | Legumes | x | 0.88 (0.80, 0.98) | 0.0 | 1.71 |  |  |  |  |  | |
|  | Rice |  | 1.28 (1.02, 1.62) | 0.0 | 1.45 |  |  |  |  |  | |
| rs1555543 | Wholegrains | x | 0.94 (0.90, 0.99) | 29.9 | 1.72 | 1:96944797 | A/C | 0.58 |  |  | |
|  | Green vegetables |  | 0.86 (0.75, 0.99) | 0.0 | 1.50 |  |  |  |  |  | |
| rs11191560 | SSB | x | 1.37 (1.05, 1.78) | 0.0 | 1.71 | 10:104869038 | T/C | 0.09 | NT5C2 | intronic, non-coding intronic | |
|  | Legumes | x | 1.73 (1.09, 2.73) | 64.8 | 1.71 |  |  |  |  |  | |
|  | SSB |  | 1.35 (1.01, 1.80) | 20.9 | 1.41 |  |  |  |  |  | |
| rs10401969 | Tea | x | 1.11 (1.02, 1.21) | 0.0 | 1.70 | 19:19407718 | T/C | 0.08 | SUGP1 | 3downstream, intronic, non-coding intronic | |
|  | Legumes | x | 1.27 (1.03, 1.57) | 0.0 | 1.63 |  |  |  |  |  | |
| rs8101064 | Processed meat | x | 0.59 (0.37, 0.92) | 24.3 | 1.70 | 19:7293119 | T/C | 0.96 | INSR | intronic, non-coding intronic | |
| rs4836133_C_G | Fruits |  | 0.94 (0.89, 0.99) | 0.0 | 1.69 | 5:124332103 | C/G | 0.09 |  |  | |
| rs9374842 | SSB |  | 1.25 (1.04, 1.51) | 24.9 | 1.69 | 6:120185665 | C/T | 0.75 |  |  | |
|  | Wholegrains |  | 0.96 (0.92, 1.00) | 3.6 | 1.37 |  |  |  |  |  | |
| rs1359790 | Rice |  | 0.69 (0.51, 0.95) | 25.4 | 1.68 | 13:80717156 | G/A | 0.26 |  |  | |
|  | Legumes |  | 0.81 (0.67, 0.99) | 31.2 | 1.42 |  |  |  |  |  | |
|  | SSB | x | 1.45 (1.01, 2.09) | 69.2 | 1.36 |  |  |  |  |  | |
|  | Legumes | x | 0.74 (0.55, 1.00) | 56.9 | 1.32 |  |  |  |  |  | |
| rs2261181 | Nuts and seeds | x | 2.62 (1.15, 5.95) | 57.7 | 1.66 | 12:66212318 | C/T | 0.11 | RPSAP52 | non-coding intronic | |
| rs7005992 | SSB | x | 1.52 (1.06, 2.19) | 57.6 | 1.66 | 8:126528955 | G/C | 0.16 | RP11-136O12.2 | non-coding intronic | |
| rs12429545 | Nuts and seeds | x | 1.61 (1.07, 2.42) | 0.0 | 1.64 | 13:54102206 | G/A | 0.13 |  |  | |
| rs543874 | SSB |  | 1.22 (1.03, 1.46) | 0.0 | 1.62 | 1:177889480 | A/G | 0.18 |  |  | |
| rs13201877 | SSB | x | 1.56 (1.06, 2.31) | 64.5 | 1.62 | 6:137675541 | A/G | 0.14 |  |  | |
| rs11717195 | Tea | x | 1.08 (1.01, 1.15) | 0.0 | 1.61 | 3:123082398 | T/C | 0.21 | ADCY5 | intronic, non-coding intronic | |
|  | Tea |  | 1.07 (1.00, 1.13) | 0.0 | 1.42 |  |  |  |  |  | |
| rs17169104 | Tea |  | 1.07 (1.01, 1.13) | 0.0 | 1.61 | 7:15883727 | C/G | 0.31 | AC006041.1 | 5upstream | |
| rs2237895 | Fish |  | 1.55 (1.06, 2.26) | 0.0 | 1.61 | 11:2857194 | A/C | 0.43 | KCNQ1 | intronic, non-coding intronic | |
|  | Rice |  | 0.59 (0.36, 0.96) | 70.5 | 1.50 |  |  |  |  |  | |
| rs12427353 | Root vegetables | x | 1.26 (1.03, 1.55) | 0.0 | 1.59 | 12:121426901 | G/C | 0.20 | HNF1A | intronic | |
| rs10767664 | Legumes |  | 0.83 (0.70, 0.98) | 21.6 | 1.59 | 11:27725986 | T/A | 0.77 | RP11-587D21.4 | intronic, non-coding intronic | |
|  | Legumes | x | 0.84 (0.71, 0.99) | 15.0 | 1.48 |  |  |  |  |  | |
| rs9881942 | Nuts and seeds |  | 0.75 (0.58, 0.97) | 0.0 | 1.57 | 3:123082416 | A/G | 0.55 | ADCY5 | intronic, non-coding intronic | |
| rs7243357 | SSB |  | 0.81 (0.68, 0.98) | 0.0 | 1.57 | 18:56883319 | T/G | 0.17 |  |  | |
| rs13233731 | Unprocessed red meat |  | 1.34 (1.03, 1.73) | 30.5 | 1.57 | 7:130437689 | G/A | 0.47 |  |  | |
| rs12286929 | Egg/egg products |  | 1.17 (1.02, 1.34) | 0.0 | 1.56 | 11:115022404 | A/G | 0.53 |  |  | |
|  | Processed meat | x | 1.13 (1.00, 1.29) | 0.0 | 1.32 |  |  |  |  |  | |
| rs11030104 | Legumes |  | 1.12 (1.01, 1.24) | 0.0 | 1.56 | 11:27684517 | A/G | 0.22 | BDNF | intronic, non-coding intronic | |
|  | Legumes | x | 1.14 (1.00, 1.29) | 3.4 | 1.35 |  |  |  |  |  | |
| rs7359397 | SSB | x | 0.84 (0.73, 0.98) | 0.0 | 1.56 | 16:28885659 | C/T | 0.38 | SH2B1 | 3downstream | |
| rs9425291 | Tea |  | 1.06 (1.01, 1.12) | 0.0 | 1.55 | 1:172312769 | G/A | 0.43 | DNM3 | intronic | |
|  | Tea | x | 1.07 (1.01, 1.13) | 0.0 | 1.50 |  |  |  |  |  | |
| rs4506565 | Coffee | x | 0.93 (0.87, 0.99) | 27.8 | 1.54 | 10:114756041 | A/T | 0.34 | TCF7L2 | intronic | |
|  | Coffee |  | 0.95 (0.91, 1.00) | 3.3 | 1.43 |  |  |  |  |  | |
| rs4607517 | Unprocessed red meat | x | 1.40 (1.03, 1.89) | 0.0 | 1.54 | 7:44235668 | G/A | 0.17 | GCK | non-coding intronic | |
| rs4804833 | Unprocessed red meat |  | 0.77 (0.61, 0.97) | 11.6 | 1.53 | 19:7970635 | A/G | 0.60 | MAP2K7 | intronic, non-coding intronic | |
|  | Green vegetables |  | 0.86 (0.75, 1.00) | 0.0 | 1.35 |  |  |  |  |  | |
| rs492400 | Fruits |  | 0.96 (0.92, 1.00) | 32.6 | 1.53 | 2:219349752 | C/T | 0.57 | USP37 | intronic, non-coding intronic | |
| rs657452 | Fish | x | 0.64 (0.42, 0.96) | 1.0 | 1.52 | 1:49589847 | A/G | 0.61 | AGBL4 | intronic, non-coding intronic | |
|  | Unprocessed red meat | x | 1.32 (1.03, 1.69) | 13.8 | 1.51 |  |  |  |  |  | |
|  | Fish |  | 0.67 (0.46, 0.97) | 0.0 | 1.47 |  |  |  |  |  | |
| rs3888190 | SSB | x | 0.85 (0.73, 0.98) | 0.0 | 1.52 | 16:28889486 | C/A | 0.38 | ATP2A1 | 3downstream, 5upstream | |
| rs2279525 | Wholegrains | x | 1.05 (1.00, 1.09) | 0.0 | 1.51 | 4:23794252 | T/C | 0.29 | PPARGC1A | 3utr, non-coding intronic | |
|  | Tea |  | 0.95 (0.90, 1.00) | 0.0 | 1.34 |  |  |  |  |  | |
| rs12885454 | Nuts and seeds |  | 1.63 (1.04, 2.54) | 46.4 | 1.50 | 14:29736838 | C/A | 0.35 | RP11-562L8.1 | non-coding, non-coding intronic | |
| rs10968576 | SSB | x | 1.19 (1.02, 1.40) | 0.0 | 1.50 | 9:28414339 | A/G | 0.29 | LINGO2 | intronic | |
|  | SSB |  | 1.15 (1.00, 1.33) | 0.0 | 1.30 |  |  |  |  |  | |
| rs11126666 | Fruits | x | 1.08 (1.01, 1.16) | 65.1 | 1.50 | 2:26928811 | G/A | 0.26 | KCNK3 | intronic | |
| rs7957197 | Root vegetables | x | 1.25 (1.02, 1.53) | 0.0 | 1.49 | 12:121460686 | T/A | 0.20 | OASL | 3downstream, intronic | |
| rs12940622 | Fermented Dairy | x | 1.09 (1.01, 1.17) | 0.0 | 1.47 | 17:78615571 | G/A | 0.43 | RPTOR | intronic, non-coding intronic | |
|  | Legumes | x | 0.90 (0.82, 1.00) | 0.0 | 1.33 |  |  |  |  |  | |
| rs7138803 | Wholegrains | x | 0.96 (0.92, 1.00) | 0.0 | 1.46 | 12:50247468 | G/A | 0.39 |  |  | |
| rs12566985 | Nuts and seeds |  | 1.33 (1.02, 1.72) | 0.0 | 1.46 | 1:75002193 | G/A | 0.57 | FPGT-TNNI3K | intronic | |
| rs9925964 | Fish | x | 0.37 (0.15, 0.93) | 67.5 | 1.45 | 16:31129895 | A/G | 0.38 | KAT8 | intronic, non-coding, non-coding intronic | |
| rs11672660 | Root vegetables | x | 0.82 (0.68, 0.99) | 0.0 | 1.45 | 19:46180184 | C/T | 0.21 | GIPR | 3downstream, 5upstream, intronic | |
| rs206936 | SSB | x | 1.22 (1.01, 1.48) | 0.0 | 1.45 | 6:34302869 | A/G | 0.21 | NUDT3 | intronic | |
| rs10423928 | Root vegetables | x | 0.82 (0.69, 0.99) | 0.0 | 1.43 | 19:46182304 | T/A | 0.21 | GIPR | 3downstream, intronic | |
| rs1558902 | Coffee |  | 1.04 (1.00, 1.08) | 1.8 | 1.43 | 16:53803574 | T/A | 0.43 | FTO | intronic, non-coding intronic | |
| rs9540493 | Egg/egg products |  | 1.16 (1.01, 1.34) | 0.0 | 1.41 | 13:66205704 | A/G | 0.55 |  |  | |
|  | Legumes | x | 0.75 (0.57, 1.00) | 66.5 | 1.32 |  |  |  |  |  | |
| rs7176058 | Fermented Dairy | x | 1.28 (1.01, 1.63) | 72.9 | 1.41 | 15:39464167 | A/G | 0.15 | RP11-624L4.1 | non-coding intronic | |
| rs1111875 | Fermented Dairy | x | 0.92 (0.86, 1.00) | 0.0 | 1.41 | 10:94462882 | C/T | 0.39 |  |  | |
|  | Fermented Dairy |  | 0.94 (0.88, 1.00) | 0.0 | 1.35 |  |  |  |  |  | |
| rs13266634 | Egg/egg products | x | 0.65 (0.44, 0.98) | 74.1 | 1.41 | 8:118184783 | C/T | 0.29 | SLC30A8 | **Coding (276, 325: R>R\| R>W)** | |
| rs9641123 | SSB |  | 1.17 (1.01, 1.35) | 1.5 | 1.38 | 7:93197732 | G/C | 0.41 | CALCR | intronic | |
| rs3802177 | Egg/egg products | x | 0.66 (0.44, 0.98) | 74.0 | 1.38 | 8:118185025 | G/A | 0.29 | SLC30A8 | 3downstream, 3utr | |
| rs13191362 | Tea | x | 0.89 (0.79, 1.00) | 13.3 | 1.38 | 6:163033350 | A/G | 0.12 | PARK2 | Intronic | |
| rs17168486 | Tea | x | 1.17 (1.01, 1.36) | 51.5 | 1.37 | 7:14898282 | C/T | 0.17 | DGKB | intronic, non-coding intronic | |
| rs731839 | Fermented Dairy | x | 1.09 (1.00, 1.18) | 0.0 | 1.37 | 19:33899065 | G/A | 0.66 | PEPD | intronic, non-coding intronic | |
| rs10203174 | Fruits | x | 1.08 (1.00, 1.16) | 37.2 | 1.35 | 2:43690030 | C/T | 0.11 | THADA | intronic, non-coding intronic | |
|  | SSB |  | 1.41 (1.00, 1.99) | 49.3 | 1.30 |  |  |  |  |  | |
| rs4256980 | Tea |  | 1.09 (1.00, 1.19) | 30.9 | 1.35 | 11:8673939 | C/G | 0.63 | TRIM66 | intronic, non-coding intronic | |
|  | Unprocessed red meat |  | 0.81 (0.66, 1.00) | 0.0 | 1.32 |  |  |  |  |  | |
| rs12779790 | Fruits | x | 0.94 (0.89, 1.00) | 39.3 | 1.34 | 10:12328010 | A/G | 0.19 |  |  | |
| rs17203016 | Fermented Dairy | x | 1.17 (1.00, 1.36) | 50.5 | 1.34 | 2:208255518 | A/G | 0.19 | AC007879.5 | non-coding intronic | |
| rs10938397 | Rice | x | 1.72 (1.01, 2.91) | 69.1 | 1.34 | 4:45182527 | A/G | 0.42 |  |  | |
| rs4846565 | Tea |  | 0.95 (0.90, 1.00) | 0.0 | 1.33 | 1:219722104 | G/A | 0.32 |  |  | |
| rs11257655 | Fruits | x | 0.95 (0.89, 1.00) | 40.8 | 1.33 | 10:12307894 | C/T | 0.22 |  |  | |
| rs10733682 | Fruits | x | 1.03 (1.00, 1.07) | 0.0 | 1.33 | 9:129460914 | A/G | 0.53 | LMX1B | 3downstream, 3utr, 5upstream | |
| rs243088 | Legumes | x | 0.91 (0.82, 1.00) | 0.0 | 1.32 | 2:60568745 | A/T | 0.46 |  |  | |
| rs7955901 | SSB |  | 1.15 (1.00, 1.31) | 0.0 | 1.32 | 12:71433293 | C/T | 0.52 | CTD-2021H9.2 | non-coding intronic | |
| rs3864041 | Root vegetables | x | 1.19 (1.00, 1.42) | 0.0 | 1.32 | 3:15185634 | T/C | 0.43 | RPS3AP53 | 3downstream | |
| rs11727676 | Unprocessed red meat |  | 0.70 (0.49, 1.00) | 0.0 | 1.31 | 4:145659064 | T/C | 0.09 | HHIP | **Coding (686: I>I)**, non-coding intronic | |
| rs10995441 | Unprocessed red meat | x | 1.32 (1.00, 1.73) | 0.0 | 1.31 | 10:64869239 | G/T | 0.77 | RNU6-543P | 5upstream | |

^1^ Multiplicative SNP-food interactions with p<0.05 are presented. Individual cross-product terms between 247 SNPs and 15 food groups were tested individually for incident type 2 diabetes (n=21,437; n_cases_=9,542) were examined with Prentice-weighted Cox proportional hazard regression adjusting for covariates as the primary analysis, with and without inclusion of BMI as a covariate: in the column of “BMI adj”, “x” indicated that BMI was included as a covariate.
^3^ The exponentiated betas are presented, indicating a ratio of two hazard ratios (HR_1_ and HR_0_) per 1 serving/day of food and the subscript indicates presence of the effect allele.
^4^– log_10_ (p-values) are presented. As the interaction estimates with p<0.05 are presented, and thus all the presented values are >log_10_(0.05), i.e. 1.301. None of the results were statistically significant when a Benjamini-Yekutieli procedure was applied to the inferences to account for the multiple tests (food items and SNPs).
^5^ Each value represents a crude, observed frequency of each effect allele (the second letter of ‘Allele’ column) in EPIC-InterAct participants.
^6^ Each gene symbol was obtained from SNPnexus (<https://www.snp-nexus.org/> accessed on October 25, 2025) (16) in which we annotated genetic information available in Ensembl database (<https://www.ensembl.org/index.html>) to each SNP. No genetic symbol indicated the SNP on an intergenic region. Multiple types of functional information indicate known transcript variants.
^7^ SSB, Sugar-sweetened beverages.

Supplemental Table 6. Genetic association results of the single nucleotide polymorphisms (SNPs) identified to have a potential interaction with one or more of fifteen food groups for incident type 2 diabetes in EPIC-InterAct case-cohort study.

See Supplemental Excel file**.**

# References

1. The InterAct Consortium. Design and cohort description of the InterAct Project: an examination of the interaction of genetic and lifestyle factors on the incidence of type 2 diabetes in the EPIC Study. Diabetologia Springer-Verlag; 2011;54:2272–82.

2. InterAct Consortium, Peters T, Brage S, Westgate K, Franks PW, Gradmark A, Tormo Diaz MJ, Huerta JM, Bendinelli B, Vigl M, et al. Validity of a short questionnaire to assess physical activity in 10 European countries. Eur J Epidemiol 2012;27:15–25.

3. Sacerdote C, Ricceri F, Rolandsson O, Baldi I, Chirlaque M-D, Feskens E, Bendinelli B, Ardanaz E, Arriola L, Balkau B, et al. Lower educational level is a predictor of incident type 2 diabetes in European countries: The EPIC-InterAct study. Int J Epidemiol 2012;41:1162–73.

4. Cooper AJ, Forouhi NG, Ye Z. Fruit and vegetable intake and type 2 diabetes : EPIC-InterAct prospective study and meta-analysis. Eur J Clin Nutr 2012;66:1082–92.

5. Aune D, Norat T, Romundstad P, Vatten LJ. Whole grain and refined grain consumption and the risk of type 2 diabetes: A systematic review and dose-response meta-analysis of cohort studies. Eur J Epidemiol 2013;28:845–58.

6. Hu E a, Pan A, Malik V, Sun Q. White rice consumption and risk of type 2 diabetes: meta-analysis and systematic review. BMJ 2012;344:e1454.

7. Afshin A, Micha R, Khatibzadeh S, Mozaffarian D. Consumption of nuts and legumes and risk of incident ischemic heart disease, stroke, and diabetes: a systematic review and meta-analysis. Am J Clin Nutr 2014;100:278–89.

8. Gijsbers L, Ding EL, Malik VS, Goede J De, Geleijnse JM, Soedamah-muthu SS. Consumption of dairy foods and diabetes incidence : a dose-response meta-analysis of observational studies. Am J Clin Nutr 2016;103:1111–24.

9. Aune D, Orat T, Romundstad P, Vatten LJ. Dairy products and the risk of type 2 diabetes : a systematic review and dose-response meta-analysis of cohort studies. Am J Clin Nutr 2013;98:1066–83.

10. Pan A, Sun Q, Bernstein AM, Schulze MB, Manson JE, Willett WC, Hu FB. Red meat consumption and risk of type 2 diabetes : 3 cohorts of US adults and an updated meta-analysis 1 – 3. Am J Clin Nutr 2011;194:1088–96.

11. Wallin A, Di Giuseppe D, Orsini N, Patel PS, Forouhi NG, Wolk A. Fish consumption, dietary long-chain n-3 fatty acids, and risk of type 2 diabetes: systematic review and meta-analysis of prospective studies. Diabetes Care 2012;35:918–29.

12. Tamze M, Virtanen J, Gaziano JM. Egg consumption and risk of type 2 diabetes: a meta-analysis of prospective studies. Br J Nutr 2016;103:474–80.

13. Imamura F, O’Connor L, Ye Z, Mursu J, Hayashino Y, Bhupathiraju SN, Forouhi NG. Consumption of sugar sweetened beverages, artificially sweetened beverages, and fruit juice and incidence of type 2 diabetes: systematic review, meta-analysis, and estimation of population attributable fraction. BMJ 2015;351:1–12.

14. Ding M, Bhupathiraju SN, Chen M, Van Dam RM, Hu FB. Caffeinated and decaffeinated coffee consumption and risk of type 2 diabetes: A systematicreview and a dose-response meta-analysis. Diabetes Care 2014;37:569–86.

15. Yang J, Mao Q-X, Xu H-X, Ma X, Zeng C-Y. Tea consumption and risk of type 2 diabetes mellitus: a systematic review and meta-analysis update. BMJ Open 2014;4:e005632.

16. Oscanoa J, Sivapalan L, Gadaleta E, Dayem Ullah AZ, Lemoine NR, Chelala C. SNPnexus: a web server for functional annotation of human genome sequence variation (2020 update). Nucleic Acids Res 2020;48:W185–92.
